# Gene Expression in the Scleractinian *Acropora microphthalma* Exposed to High Solar Irradiance Reveals Elements of Photoprotection and Coral Bleaching

**DOI:** 10.1371/journal.pone.0013975

**Published:** 2010-11-12

**Authors:** Antonio Starcevic, Walter C. Dunlap, John Cullum, J. Malcolm Shick, Daslav Hranueli, Paul F. Long

**Affiliations:** 1 Section for Bioinformatics, Department of Biochemical Engineering, Faculty of Food Technology and Biotechnology, University of Zagreb, Zagreb, Croatia; 2 Centre for Marine Microbiology and Genetics, Australian Institute of Marine Science, Townsville, Australia; 3 Department of Genetics, University of Kaiserslautern, Kaiserslautern, Germany; 4 School of Marine Sciences, University of Maine, Orono, Maine, United States of America; 5 The School of Pharmacy, University of London, London, United Kingdom; University of Canterbury, New Zealand

## Abstract

**Background:**

The success of tropical reef-building corals depends on the metabolic co-operation between the animal host and the photosynthetic performance of endosymbiotic algae residing within its cells. To examine the molecular response of the coral *Acropora microphthalma* to high levels of solar irradiance, a cDNA library was constructed by PCR-based suppression subtractive hybridisation (PCR-SSH) from mRNA obtained by transplantation of a colony from a depth of 12.7 m to near-surface solar irradiance, during which the coral became noticeably paler from loss of endosymbionts in sun-exposed tissues.

**Methodology/Principal Findings:**

A novel approach to sequence annotation of the cDNA library gave genetic evidence for a hypothetical biosynthetic pathway branching from the shikimic acid pathway that leads to the formation of 4-deoxygadusol. This metabolite is a potent antioxidant and expected precursor of the UV-protective mycosporine-like amino acids (MAAs), which serve as sunscreens in coral phototrophic symbiosis. Empirical PCR based evidence further upholds the contention that the biosynthesis of these MAA sunscreens is a ‘shared metabolic adaptation’ between the symbiotic partners. Additionally, gene expression induced by enhanced solar irradiance reveals a cellular mechanism of light-induced coral bleaching that invokes a Ca^2+^-binding synaptotagmin-like regulator of SNARE protein assembly of phagosomal exocytosis, whereby algal partners are lost from the symbiosis.

**Conclusions/Significance:**

Bioinformatics analyses of DNA sequences obtained by differential gene expression of a coral exposed to high solar irradiance has revealed the identification of putative genes encoding key steps of the MAA biosynthetic pathway. Revealed also by this treatment are genes that implicate exocytosis as a cellular process contributing to a breakdown in the metabolically essential partnership between the coral host and endosymbiotic algae, which manifests as coral bleaching.

## Introduction

Reef-building corals (Anthozoa: Scleractinia) that typically inhabit the nutrient-poor and shallow waters of tropical marine ecosystems accommodate dense populations of endosymbiotic dinoflagellates of the genus *Symbiodinium* (known colloquially as zooxanthellae), which is divided into distinct sub-generic lineages (clades A–D). This phototrophic association allows the release of organic carbon produced by the algal partner for coral nutrition while metabolic wastes from the animal are recycled to fertilize algal photosynthesis [1]. Because the dinoflagellates reside within endodermal cells of the host animal, coral tissues must be transparent to facilitate the penetration of downwelling light required for algal photosynthesis. In clear shallow waters this entails concurrent exposure of vulnerable molecular sites in the partners to potentially damaging wavelengths of solar ultraviolet radiation (UVR). In addition, photosynthetic endosymbionts typically release more oxygen than the symbiosis is able to consume in respiration, so that animal tissues are hyperoxic when illuminated, with pO_2_ often exceeding 250% air saturation during routine exposure to normal daytime irradiances [2]. The synergetic stress of UV exposure and hyperoxia has the potential to cause photooxidative damage to the symbiosis via the photochemical production of cytotoxic reactive oxygen species (ROS) [3] that are produced also during normal mitochondrial respiration [4]. Biochemical defences against photochemical damage from direct exposure to solar UVR and indirectly from ROS production enhanced by UVR include the biosynthesis of UV-absorbing compounds (sunscreens), cellular reductants and antioxidants, and the elaboration of antioxidant enzymes (reviewed in [5], [6]). These cellular defences in corals and other marine invertebrates are often induced under conditions of oxidative stress, including UV exposure [7].

The cellular responses of the coral holobiont to thermal stress alone or in combination with other environmental stressors (including UVR), sometimes manifested as bleaching (loss of endosymbionts from the host and/or by damage to photosynthetic pigments), continue to be elucidated [8]. Coral bleaching has been studied mostly at the physiological level by detecting the progressive loss of symbiotic algae or by measuring changes in the integrity and performance of their photosynthetic apparatus [9], [10]. At the molecular level, emerging genomic sequencing [11] and cDNA technologies enable the detection of differentially expressed genes of the coral holobiont transcribed under altered physiological states, which offer exceptional promise as tools in marine symbiology [12], [13], [14], [15], [16], [17], [18]. Such methods include differential display PCR (ddPCR), representational difference analysis (RDA), serial analysis of gene expression (SAGE), RNA arbitrarily primed PCR, real-time PCR (RT-PCR) and hybridization to cDNA microarrays. These methods, however, are frequently inefficient in detecting transcripts that are low in abundance and often generate high yields of false-positive results [19]. In contrast, the PCR-based suppression subtractive hybridization (PCR-SSH) technique is a powerful method that overcomes the problem of differences in mRNA abundance by using a cDNA hybridization procedure that normalizes sequence abundance by kinetic suppression in the PCR subtractive step to afford greater than 10^3^-fold enrichment of rare sequences [20].

Herein we use the PCR-SSH method to examine regulation of photoprotective genes in the coral *Acropora microphthalma* on exposure to elevated solar radiation. A cDNA library of subtracted and differentially expressed genes was created for sequencing and gene annotation by transplanting a single colony of *A. microphthalma* from a mid-irradiance habitat at a depth of 12.7 m to near-surface solar irradiance. This treatment was intended to provide a complete differential gene-expression profile of the response of the individual coral symbiome to enhanced UV/visible light exposure, including transcripts differentially expressed in response to sunlight-induced oxidative stress and possibly the stress-response genes implicated in coral bleaching. Our primary interest, however, was to reveal genes of the coral symbiome responsible for the biosynthesis of UV-protective, mycosporine-like amino acids (MAAs).

MAAs comprise a family of some 20+ small, colourless, cytosolic compounds containing either cyclohexenone or cyclohexenimine, UV-absorbing chromophores conjugated with the nitrogen substituent of an amino acid or imino alcohol. MAAs have been isolated from marine cyanobacteria, unicellular algae and intertidal seaweeds, as well as a wide range of sessile invertebrates having diverse photosymbionts, including symbiotic corals (reviewed by [5]). MAAs identified in the coral *Acropora microphthalma* [7] are given in Figure S1. Specific inhibitors of the shikimic acid pathway block accumulation of MAAs, suggesting that MAA biosynthesis in the eukaryotic partnership of corals proceeds from a shikimate branch point thought to be at 3-dehydroquinate [21], [22], [23], [24]. MAAs in phototrophic symbioses are expected to be produced by the dinoflagellate partner, or obtained from the diet, because metazoans reputedly lack the central pathway to synthesize shikimate-derived ‘essential’ aromatic amino acids, although we have recently challenged the universality of this traditional view [25]. MAAs have long been recognised as sunscreens in coral symbiosis [26], yet the enzymes essential to their biosynthesis in this symbiosis have not been isolated and the genes encoding these enzymes are yet to be described.

## Results

### Annotation of cDNA sequences derived from *A. microphthalma* exposed to high solar irradiance

The PCR-SSH cDNA library of genes up-regulated by the coral *A. microphthalma* contained 142 sequenced clones with inserts of a median sequence length of 396 bp (dbEST database accessions: GR212027–GR212168; Table S1). The translated DNA sequences were used for searches (blastx) against the non-redundant protein database (nr) at NCBI, but this failed to yield any significant matches. This is probably due to a combination of the short sequence lengths and the fact that because corals are evolutionary distant from most of the species contributing to the protein database. We, therefore, developed a novel strategy to exploit the greater evolutionary diversity present in EST databases. In a first step, an EST database was searched with the 142 sequences using a Smith-Waterman algorithm for increased sensitivity. In a second step, the matching EST sequences were used for searches. Because many EST sequences are longer than those in the coral cDNA library, this would extend the sequences into neighbouring regions that might yield matches. In addition, longer sequences yield higher scores and lower e-values, which increase the chance of aligning significant matches.

Smith-Waterman analysis of the 142 sequences used the high-performance Sencel Paralign application (http://www.sencel.com/) for local sequence alignments applied a calculated e-value of ≤10^−2^, equivalent to the value used successfully for the annotation of a complex marine microbial metatranscriptome [30]. About 48% of our sequences produced matches in the EST database (68/142) and there were usually multiple hits for each sequence (2429 ESTs for 68 cDNA clones; http://bioserv.pbf.hr/Supplementary_Data/File1). This was not unexpected given that the cDNA sequences were smaller in length than the ESTs. However, because so many of the ESTs in the EST database proved to be contaminated by either vector or sequencing primers (an unsuspected impediment to others using the EST database), we cleaned the matching 2429 ESTs and re-ran the analysis to yield 1782 validated EST alignments (http://bioserv.pbf.hr/Supplementary_Data/File2) for annotation. Since an e-value of ≤10^−2^ was used as a cut-off criterion for the first Smith-Waterman analysis, this same value was used also in the second analysis (http://bioserv.pbf.hr/Supplementary_Data/File3). However, the two results obtained from using identical e-values were not directly comparable because the first analysis searched the entire EST database to match against our sequenced cDNA library, whereas the second analysis searched only against the 1782 validated EST alignments. To reach a consensus between the two analyses so that ESTs selected by alignment to vector or primer sequences could be rejected, only ESTs that matched the same cDNA clones in both analyses were deemed appropriate for further consideration. This gave only 12 clones matching 305 ESTs, suggesting that the other 130 cDNA sequences in the library either were too short to give a significant match to known ESTs or that the sequences were novel (http://bioserv.pbf.hr/Supplementary_Data/File4.rar). This seemingly low rate is nevertheless better than results of a translated BLAST search against protein databases, which failed to yield any significant matches and was the stimulus for us to develop our novel annotation strategy.

### Profile of gene expression in *A. microphthalma* exposed to high solar irradiance

**Figure 1:**
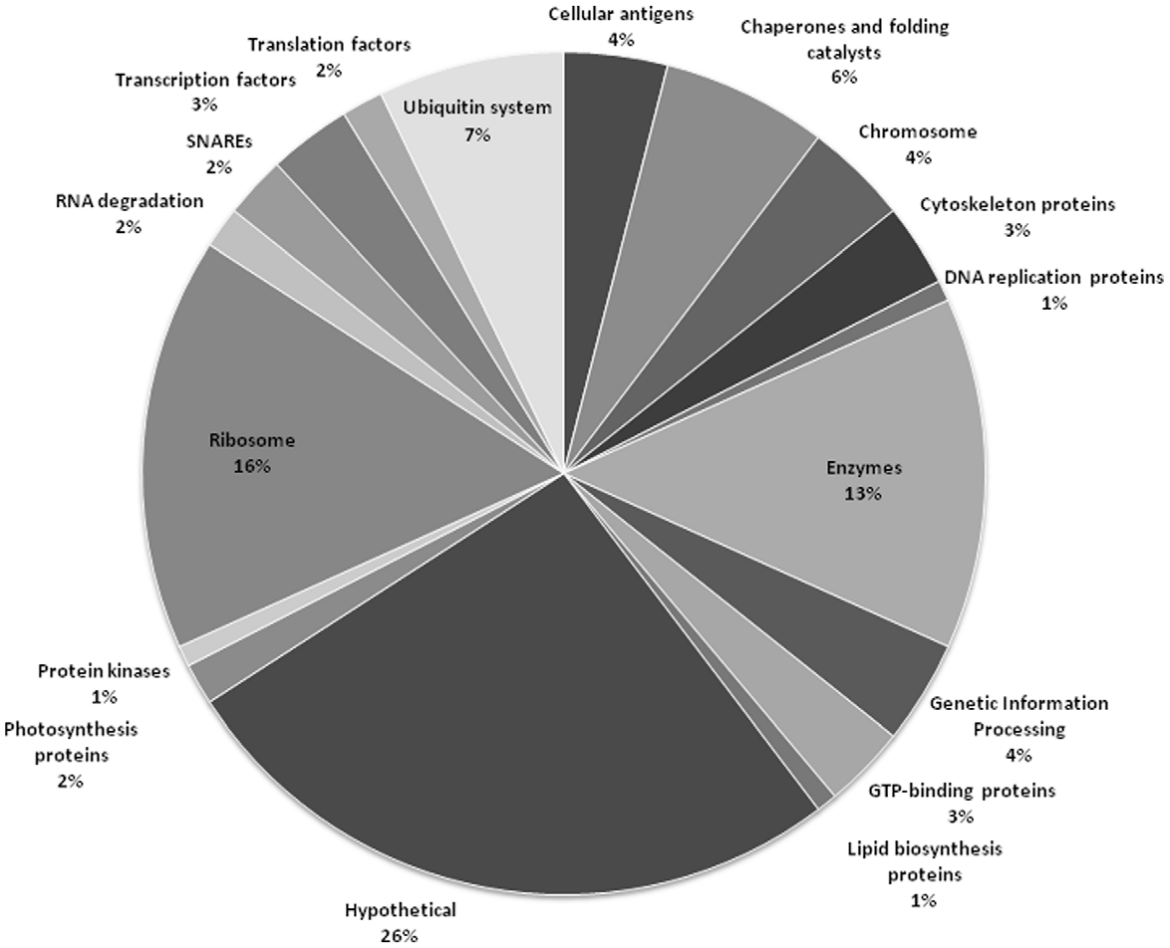
Mapping of the percentage contribution of eukaryotic transcripts differentially expressed by the *Acropora microphthalma* symbiome induced by high levels of solar irradiance to general protein families using the KEGG BRITE hierarchical classification scheme.

In the next annotation step, the 305 EST sequences resolved by Smith-Waterman analyses were used to search the non-redundant NCBI nucleotide (nr/nt) database using tBLASTx for protein sequence comparisons. The parameters chosen were the same as those in the previous steps (i.e., a calculated e-value of ≤10^−2^), which gave an output (http://bioserv.pbf.hr/Supplementary_Data/File5) that was parsed to select for the highest scoring alignment to each EST. This resulted in 131 EST matches against the 12 significant clones (Figure S2). Among these 131 sequences, 33 could be matched only to sequences of hypothetical proteins of unknown function, whereas the remaining 98 sequences could be matched to proteins having assigned potential functions. We repeated the analysis by comparing the 305 ESTs against annotated sequences of the GenomeNet database available at the KEGG Automatic Annotation Server (KAAS). This allowed 30/305 ESTs to be assigned directly by exact match to corresponding nt annotations (Figure 1 and Figure S2).

KEGG BRITE classification of translated cDNA sequences obtained from the coral symbiome in response to high solar irradiance gave 18 families of proteins; the largest group after the hypothetical proteins (33/131) were associated with protein synthesis (31/131), including the synthesis of ribosomal protein and those proteins serving as transcription and translation factors. Metabolic enzymes, which included protein kinases and those involved in lipid metabolism, constituted the next largest group (19/131), followed by genes that direct nucleic acid synthesis, turn-over and repair and those encoding the histone template of chromosomal structure (14/131). A range of proteins implicated in cytoskeletal arrangement are those involved in protein folding and vesicular transport (11/131). Genes encoding proteins of the ubiquitin system, especially proteasome formation and ubiquitin binding (9/131), were also differentially expressed, as were genes associated with immunity but of unknown cellular function (5/131). Conspicuous are genes of the algal symbiont that encode the LHCB1-like chlorophyll light-harvesting complex II protein of photosynthesis (2/131) transcribed in response to high irradiance. Molecular chaperone and antioxidant response genes (3/131) encoding heat shock protein 90 and a thioredoxin enzyme were also identified.

Genes encoding proteins not previously implicated in the response of coral stress could also be annotated from the cDNA library, notably a synaptotagamin-like protein (3/131) that is the calcium-ion sensing actuator of vesicular exocytosis primed for release by SNARE-complex fusion assembly at the cytoplasmic membrane [31]. There were also sequences similar to the small Ran GTPase proteins (4/131) that operate by GDP/GTP cycling to direct protein/RNA transport between the cytoplasm and nucleus during interphase [32] and for regulation of mitotic spindle assembly, DNA replication and nuclear envelope assembly [33]. Ran nuclear proteins are members of the Ras superfamily of GTPases, of which the Rab and Arf family members mediate vesicular transport in exocytosis [34].

### Assignment of MAA biosynthetic genes expressed in *A. microphthalma* exposed to high solar irradiance

**Figure 2:**
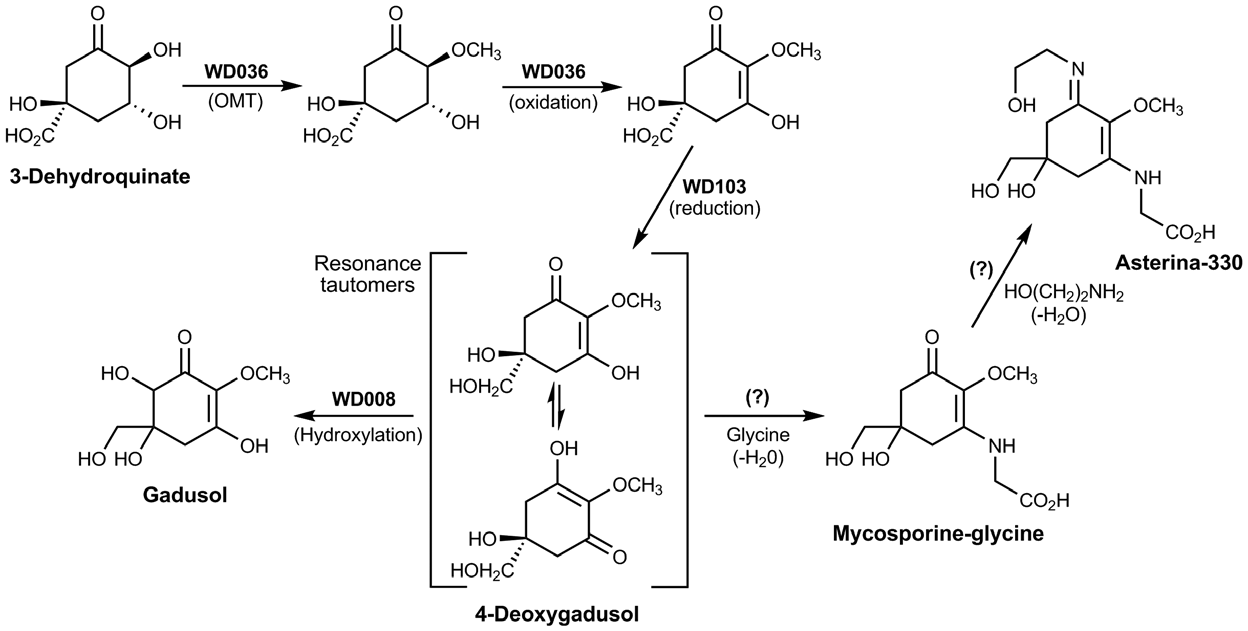
Proposed biosynthetic scheme and putative genes leading to the formation of 4-deoxygadusol, based on genes annotated from a differentially expressed cDNA library from the coral *Acropora microphthalma* following experimental low-to-high light exposure on the Great Barrier Reef.

We assumed an MAA biosynthesis route shown in Figure 2. Our EST matching approach by nucleotide sequence analysis or by KEGG BRITE protein classification did not yield directly any strong candidate genes likely involved in MAA biosynthesis. Alternatively, candidate protein sequences were extracted from the UniProt database using keywords describing the enzyme functions required for MAA biosynthesis (Figure 2). Then, using the Smith-Waterman algorithm, the functional protein sequences matched against the 142 cDNA sequences gave candidates for the four hypothetical enzyme activities predicted for MAA biosynthesis (Table S2).

Biosynthesis of the expected MAA-precursor 4-deoxygadusol (4-DG) from 3-dehydroquinate (DHQ), an intermediate in the shikimic acid pathway, requires O-methylation at C2-OH, dehydrogenation at C2–C3 and reduction of the C5 carboxylate substituent to an alkyl hydroxyl group (Figure 2). Candidate WD036 gave sequence alignment with both O-methylation and dehydrogenation enzymes, the first two steps predicted for MAA biosynthesis to follow the 3-dehydroquinate branch-point from the shikimate pathway. These results must be treated with caution because the alignments are not to regions of the UniProt enzymes of known functional significance. However, when the 242 matching amino acids from the UniProt enzyme were used for a BLAST search against the non-redundant protein database (nr) at NCBI, the best 5000 significant hits (e-values<2×10^−21^) were to sequences annotated as dehydrogenases (or in a few cases of unidentified function). Similarly, the 51 amino acid matching region of the *O*-methyltransferase enzyme only gave BLAST hits (e-values<10^−3^) to *O*-methyltransferases and a few cases of non-identified function. This reinforces the idea that WD036 may be associated with such activities.

Biosynthetic completion of 4-deoxygadusol requires reduction of COOH to CH_2_OH at C5, which matched the candidate sequence WD103. Coral MAAs have not as yet been identified with hydroxylation at C6 (from the tautomeric C4 and C6 positions of 4-deoxygadusol) since the MAA precursor is expected to be 4-deoxygadusol, rather than the C6-hydroxylated gadusol. Nevertheless, annotation of our subtracted hybridization sequences identified WD008 as a putative hydroxylase appropriate to gadusol biosynthesis. Similarly, as for the first two enzyme activities, 92 and 40 amino acid regions of the UniProt enzymes around the matches to WD103 and WD008 respectively were used for BLAST searches against the NCBI protein database (nr) and did not show any significant matches to other classes of proteins. The assignment of candidate MAA biosynthetic genes and other relevant genes induced as an acute stress response to bright sunlight are listed in Table 1. Genes encoding the ligase enzymes required to catalyse the condensation reactions downstream of 4-deoxygadusol to complete the synthesis of mycosporine-glycine and its many imino-derivatives, such as palythine, asterina-330, palythinol and palythene (Figure S1), were not identified in the coral symbiome from our cDNA library.

**Table 1:** Putative annotation of genes associated with elements of photoprotection and bleaching in *A. microphthalma* exposed to high solar irradiance.

Cellular function	Candidate genes	GenBank accession numbers	Matching alignment UniProt or EST accession number	Putative protein function
MAA biosynthesis	WD036WD137	GR212053GR212140	Q94AI4Q3JSI4Q8I555C7TQP1	O-Methylation at C2-OH; dehydrogenation at C2-C3
	WD103	GR212113	C4B488	Reduction of COOH to CH_2_OH at C5
	WD008	GR212031	Q1LX59	Hydroxylation at C6
Photosynthesis	WD047^1^WD151	GR212064GR212152	EG551345EG551245	LHCB1-like light-harvesting protein
Antioxidant response	WD029^2^	GR212046	EG551286	Thioredoxin
Stress response	1WD047	GR212064	AJ927897	Heat shock protein Hsp90
Exocytosis	WD057WD095^3^	GR212074GR212105	CJ456304CJ456304	Ca^2+^-Binding synaptotagmin-like protein effector of SNARE assembly exocytosis
Cellular Transport	WD029^2^WD035WD095^3^WD127	GR212046GR212052GR212105GR212131	AJ927925AJ927897AJ927925AJ927925	Ran family of Ras-like GTPase nuclear transport regulators

1 WD047 shares 27.8% amino acid sequence identity to EST EG551345 and 24.4% amino acid sequence identity to EST AJ927897.

2 WD029 shares 29.9% amino acid sequence identity to EST EG551286 and 31.3% amino acid sequence identity to EST AJ927925.

3 WD095 shares 28% amino acid sequence identity to EST CJ456304 and 29.7% amino acid sequence identity to EST AJ927925. At this level of annotation, it is not possible to differentiate between multiple hits to different ESTs since the cDNA sequences were smaller in length than the ESTs.

### Genetic origin of putative MAA biosynthetic sequences

Results from PCR amplification using sequence-specific primers for sequences WD036, WD103 and WD008 gave amplicons of the expected size for DNA extracted from sperm of the congeneric coral *A. millepora* but, surprisingly, not for DNA obtained from cultured dinoflagellates of clade D (Figure S3).

## Discussion

### Annotation of genes expressed in *A. microphthalma* exposed to high solar irradiance

To identify genes induced by high solar irradiance, a PCR-based, cDNA subtractive hybridization (PCR-SSH) strategy was used to generate a cDNA library of genes up-regulated by the eukaryotic consortia of the *A. microphthalma* symbiome. Although the PCR-SSH method has the important advantage of detecting differentially expressed genes occurring at low transcript levels, the lengths and quality of the sequences are lower than alternative less sensitive methods. The taxa involved (corals and dinoflagellates) are not well represented in genomic sequence databases, which renders ineffective the simple standard bioinformatics analyses using *in silico* translation and BLAST searches against protein databases. We therefore developed an unconventional strategy for sequence annotation. In the initial stage, a search was conducted to match our cDNA sequences against EST databases using the Smith-Waterman algorithm, which although slow, is the most efficient method for aligning nucleotide sequences. Careful choice of the algorithm parameters was necessary to match EST sequences of significant size. The use of DNA sequence comparisons for poor-quality sequences has the advantage that frame-shift artefacts arising from sequencing errors scarcely disturb the performed alignment. Our annotation method allowed 33% more ESTs to be assigned a potential function compared to assigning function using the publicly available KAAS server. There were sometimes conflicting annotations when a cDNA sequence gave multiple hits to more than one EST. Because we were interested in providing a broad overview of the response of the coral symbiome to light-induced stress in the absence of corroborating physiological data, we used the KEGG BRITE hierarchical classification of protein families to assign each of the 131 ESTs to a broad category of biochemical function (Figure 1 and Figure S2).

### Assignment of putative MAA biosynthetic genes

Our examination of genes differentially expressed by high solar irradiance yielded cDNA sequences encoding enzyme families corresponding to the expected pathway for the biosynthesis of UV-protective MAAs (Figure 2), which are listed in Table 1. The candidate sequence WD036 gave, to our surprise, an alignment to enzymes that catalyse both the *O*-methylation and dehydrogenation steps of MAA biosynthesis expected to follow the 3-dehydroquinate branch point from the shikimate pathway [24]. Completing the synthesis of the 4-deoxygadusol precursor is a candidate sequence matching WD103 for reduction of the C5 carboxylate group. The combination of their sunlight-induced expression and expected biochemical function makes these genes, listed in Table 1, attractive candidates for involvement in MAA biosynthesis. Future work to obtain the full sequence of WD036 and WD103 will determine whether WD036 does indeed encode a novel bifunctional fused enzyme and WD103 as a carboxylic acid reductase in MAA biosynthesis. Additionally, sequence WD008 matched a putative hydroxylase enzyme appropriate to the biosynthesis of gadusol, an important antioxidant known from *Artemia* cysts and fish eggs (reviewed in [5]), but a metabolite as yet to be described in coral symbiosis. These assignments, however, must be considered hypothetical until the biochemical functions of our putative pathway genes, and any other potential gene candidates in our dataset (e.g. WD137 as an alternative *O*-methyltransferase/dehydrogenase encoding gene; see: http://bioserv.pbf.hr/Supplementary_Data/S6.rar) have been verified experimentally.

Very recently MAA biosynthesis genes have been described from free-living cyanobacteria [35]. The biosynthetic pathway uses a precursor derived from the pentose phosphate pathway rather than the shikimate pathway, but it is striking that the first enzyme 2-*epi*-5-*epi*-valionate synthase (EVS) is so closely related to 3-dehydroquinate synthase that it had been mistakenly annotated in many cyanobacterial genomes. The neighbouring gene encodes an *O*-methyltransferase. A BLAST search using the EVS protein sequence from *Nostoc punctiforme* ATCC 29133 was used for a BLAST search of cnidarian and *Symbiodinium* symbiont ESTs at NCBI and other publically available cnidarians databases, but no significant hits were found; the databases contain about 100,000 sequences derived from corals in a total of about 630,000 ESTs.

### Genetic origin of putative MAA biosynthetic sequences

There is, as yet, no evidence for the presence of an EVS enzyme in coral symbiosis. If MAAs were synthesised in this symbiosis using the same pathway as in cyanobacteria, then the synthesis should be independent of the shikimate pathway. It is conceivable that the inhibition of MAA synthesis by the shikimate pathway inhibitor glyphosate [22] occurs by indirect mechanisms, but this probably would not explain the observed rapid effect of biosynthetic inhibition. Initial results from PCR amplification using sequence-specific primers are remarkable because they suggest that enzymes to complete MAA biosynthesis are located not in the algal (dinoflagellate) endosymbiont as would be expected from MAAs having a precursor from the iconic shikimic acid pathway known in plants and microorganisms, but rather in the coral host. That is, essential MAA-biosynthetic genes identified in the symbiome of *A. microphthalma* appear lacking in cultured *Symbiodinium* symbionts (clade D) but were found in the spermatozoa of the congeneric coral *A. millepora* (Figure S3). Only symbiotic dinoflagellates of clade A synthesize MAAs on exposure of cultures to UVR [36], although *Symbiodinium* spp. of all clades do contain MAAs when in symbioses with cnidarians [37]. It was not, therefore, surprising that DNA from dinoflagellates of clade D lacks our putative MAA biosynthetic genes (Figure S3), whereas the clearly evident gene PCR products from DNA of coral sperm from congeneric *A. millepora* were unexpected. Consequently, complete MAA biosynthesis is predicted to be a “shared metabolic adaptation” of symbiosis [25], with the required biochemical intermediates from the shikimic acid pathway emanating from the dinoflagellate partner and final steps of the biosynthesis occurring in the host.

It does seem surprising for MAAs to be synthesised by the animal host rather than by the algal endosymbiont, especially because atmospheric, biogeochemical, and evolutionary considerations suggest that MAA biosynthesis arose in the cyanobacteria [38] and was passed to eukaryotic algae via the plastids that originated from an ancestral cyanobacterium. Thus, many free-living marine algae and cyanobacteria produce MAAs for UV protection, including the cyanobacterium *Anabaena variabilis*, which is a favoured model for studying MAA biosynthesis [39], [40]. Indeed, the genomes of many known cyanobacterial MAA-producers contain a fusion gene for DHQ synthase and *O*-methyltransferase (Table S3). A sequence alignment of active site residues clearly shows these to be DHQ synthases and not EVS, however, in some cases a fusion between EVS and *O*-methyltransferase might also be possible (Table S4). Indeed, a bifunctional DHQ-OMT synthase gene was detected also in the genome of the asymbiotic sea anemone, *Nematostella vectensis* [25]. Thought to be acquired from algae via horizontal gene transfer, the fused gene in the genome of *N. vectensis* aligned closely with that of the dinoflagellates *Oxyrrhis marina* and *Heterocapsa triquetra*, both from families known to produce MAAs [41], and so implicates DHQ as the shikimate branch-point of MAA biosynthesis in these dinoflagellates.

### Genes involved in the oxidative stress response

Contrary to expectation, acutely exposing a colony of *A. microphthalma* to strong levels of solar radiation for 1.5 days during winter, and avoiding the interference of summertime thermal stress, did not elicit in either the endosymbiont or the host the transcription genes encoding the key antioxidant enzymes superoxide dismutase, catalase, ascorbate peroxidase, or glutathione peroxidase. Clearly expressed, however, were thioredoxin (a hydroperoxide reductase that inhibits radical-chain lipid peroxidation) and the ubiquitous chaperone heat shock protein, Hsp90.

Antioxidant enzyme activities are typically enhanced in anthozoans and their resident dinoflagellates in response to natural and long-term (days to weeks) experimental conditions that promote oxidative stress [7], [42], [43], but enhanced gene transcription for key antioxidant enzymes was not detected in our PCR-SSH experiment where acute photic stress lasted for only 1.5 days, nor was it reported in another coral PCR-SSH experiment after 4 h of acute exposure to elevated temperature and artificial UVR [12]. Microarray cDNA methods of analysis did detect enhanced transcription of genes in coral encoding antioxidant enzymes, including catalase, after ten days of thermal stress [44]. Up-regulation of MnSOD was detected in the coral host after at least nine days of thermal stress [17] and increased concentrations of CuZn-SOD were observed using an antibody for this protein in corals following five days of heat stress [45]. In contrast, genes involved in protecting cells against oxidative stress showed no response in aposymbiotic coral larvae on short term exposure (3 and 10 hours) to hyperthermal stress [46]. Taken together, these results suggest that the expression of genes encoding key antioxidant enzymes is not enhanced by the immediate effects of short-term light or thermal stress and, consistent with our results, several days or more of duress may be required for transcriptional activation of these enzymes.

### Genes involved in the coral bleaching response

Initiation of the molecular events leading to coral bleaching has been attributed to the light-induced inhibition of photosystem II that, in combination with elevated temperature, damages the D1 protein in the PSII reaction centre (reviewed in [47]) or inhibits *de novo* synthesis of the chlorophyll-peridinin light-harvesting protein complex of dinoflagellates [48], subsequently producing reactive oxygen species (ROS) formed by excess electrons from the over-reduced photosynthetic electron transport chain [6]. Consistent with accumulated damage, light-induced gene transcription of photosynthetic proteins in our PCR-SSH experiment with *A. microphthalma* did not reveal enhanced transcription of the photosynthetic D1 protein, suggesting that its turnover may not be regulated at the transcriptome, at least in this experiment without accompanying thermal stress. Instead, multiple cDNA sequences were cloned that encode the light-harvesting chlorophyll-binding protein in PSII, which is usual in light-regulated transcription of chloroplastic genes [49].

Five separate mechanisms have been invoked to explain the loss of algal symbionts during coral bleaching caused by excessive light and heat: in situ degradation and digestion of symbionts, symbiont exocytosis, host cell detachment, host cell necrosis, and host cell and symbiont apoptosis [8]. Stress-induced generation of ROS is thought to actuate an innate immune response by the host to evoke apoptosis, a pathway in vogue to explain the molecular response of corals leading to bleaching [50], [51]. In this process, ROS induce the transcription factor NF-κB, which can activate apoptosis directly or indirectly by the induction of nitric oxide synthase to activate the pro-apoptotic transcription factor p53. This in turn promotes transcription of downstream caspases, which are the proteolytic enzymes responsible for apoptosis. No transcripts encoding any of these elements were detected in our differentially expressed cDNA library.

We found, instead, the transcription of genes associated with vesicular transport and cytoskeletal rearrangement that enable phagosomal exocytosis (Table 1). These were revealed in two clones across three ESTs encoding the Ca^2+^-sensing synaptotagmin-like protein effector of coral SNARE assembly proteins for vesicular fusion at the cell membrane [52]. Sequences of four clones encode proteins most closely related to the Ran family of Ras-like GTPase regulators of protein and RNA transport between the cytoplasm and nucleus [53]. Under conditions of acute stress, promotion of Ran-GTPase transcription may occur to accommodate greater protein/mRNA trafficking from the enhanced nuclear activity of RNA production for protein synthesis, as indicated by a pronounced increase in the RNA/DNA ratio measured in the light acclimation [54] and bleaching response of corals [55]. Recently, Ran GTPase has been shown to interact with cytoskeletal myosin to direct phagocytosis of viral pathogens in a marine invertebrate [56]. Yet, although Ran proteins have common sequence motifs with that of the Rab family of GTPase transport proteins well studied in phogocytosis [34], it is unclear if, or how, Ran-GTPase proteins may be involved directly in phagosomal exocytosis during cellular processes of coral bleaching. A discussion supporting vesicular transport and exocytosis in coral bleaching based on contemporary evidence is given in Text S1 (*highly recommended reading*).

Our PCR-SSH experiment generating a cDNA library from transcripts differentially expressed by the eukaryotic component of the coral symbiome, together with a novel comprehensive strategy of bioinformatics analyses, successfully identified putative photoprotective MAA-biosynthetic genes, antioxidant response genes and cellular elements of coral bleaching induced in *A. microphthalma* on exposure to high levels of solar irradiance. Although this treatment did not provide evidence for the entire MAA biosynthetic pathway in the symbiome of *A. microphthalma*, the procedure did identify hypothetical genes encoding enzymes having catalytic specificities that matched exactly the expected structural transformations required for the biosynthetic conversion of 3-dehydroquinate (DHQ) to 4-deoxygadusol, the direct precursor of the MAAs. Furthermore, molecular evidence from PCR amplification of sequences assigned to this pathway surprisingly indicates that genes encoding the post-DHQ pathway leading to MAAs in the coral symbiome are located in the coral host, not in the resident dinoflagellates, so that the biosynthetic pathway is a shared adaptation between the algal and invertebrate partners. The genetic sequences thus obtained from the differentially expressed transcriptome of *A. microphthalma* provide a useful molecular template for full-length gene validation required for elucidating whether MAA biosynthesis in coral symbiosis originates from a branch point of the shikimic acid pathway and, therefore, different from some free-living, prokaryotic, MAA-producing photoautotrophs where MAA biosynthesis is derived from the pentose-phosphate pathway [35]. If this were the case, then the evolutionary divergence of DHQ synthase and EVS from a common ancestor and how DHQ synthase is coupled with *O*-methyltransferase in many free-living producers and in invertebrate-algal symbiosis would be of considerable value to better understand the UV-photophysiology of marine organisms adapted to shallow-water habitation.

The sequence library also revealed transcription of the calcium-sensing synaptotagmin activator of SNARE protein assembly of vesicular trans-membrane trafficking and Ran-like GTPase transport proteins, consistent with an emerging paradigm of phagosomal exocytosis as the cellular mechanism of the release of algal endosymbionts from host tissues in coral bleaching. Although empirical validation is required to confirm the precise cellular function encoded by these genetic assignments, our bioinformatics annotation of differentially expressed genes in *A. microphthalma* offers new leads for examining the stress response of a coral symbiome to environmental conditions that may cause coral bleaching. Furthermore, our novel bioinformatics approach may prove useful in systems biology for the transcriptional analysis of other exotic metagenomes where established sequence identities are low, or for annotation of short read-lengths from high-throughput sequencing methods that are difficult to interpret.

## Materials and Methods

### Collection of *Acropora micropthalma* and exposure to high solar irradiance

A colony of *Acropora microphthalma* [27] that harboured clade D1 dinoflagellates (ITS1 standard: GenBank EU024793), as determined by ITS1 single stranded conformational polymorphism (ITS1-SSCP) analysis [28], [29], was collected on 4 August 2005 at low tide from a depth of 12.7 meters at Davies Reef, in the central Great Barrier Reef, Australia. The UV-absorption spectrum of the methanolic extract of a coral fragment indicated only low levels of MAAs absorbing in the expected range, 310–360 nm (e.g., Figure 4 in [26]). Part of the coral specimen (the ‘river’ was flash frozen in liquid nitrogen. The remainder of the specimen was immersed in an open shallow bin of running ambient seawater (23.4–23.8°C) under full sunlight (50–1950 µE⋅m^−2^⋅s^−1^ surface PAR) on a mild winter day (21.6–22.6°C) aboard the RV Cape Ferguson while anchored in calm water at Davies Reef. After a day and a half of exposure, the UV-absorption spectrum of the methanol extract of a fragment of the light-exposed coral clearly showed an increase in absorbance at the waveband attributed to MAA production, depicting a change in biosynthetic gene expression (Figure S4). During this treatment, the sun-exposed surfaces of the coral colony paled noticeably, indicating a partial loss of algae in the onset of light-induced bleaching. An additional sample from the light-exposed coral (the ‘ester’ was flash frozen in liquid nitrogen for the PCR-SSH reaction.

### RNA isolation

Coral tissue was removed from the frozen skeletons of both the ‘Driver’ and ‘Tester’ specimens by blasting with a solution of 0.02% SDS (w/v) in sterile sea water under a jet of air. The tissue was homogenized and filtered through a 200 µm screen and a 20 µm filter to remove skeletal fragments and cell aggregates. The cells were collected by centrifugation (3000 rpm for 15 min at 4°C) then washed in sterile, ice-cold phosphate buffer (pH 7.4) before being suspended in 500 µl RNeasy RTL lysis buffer (Qiagen Pty Ltd., Doncaster, VIC, Australia) without added mercaptoethanol. Handling and processing time did not exceed 15 min. Subsequently, the cells were lysed in buffer by sonication using 3×30 sec blasts with cooling in ice water. Total RNA of the holobiont was extracted from both ‘Driver’ and ‘Tester’ samples with phenol∶chloroform∶isoamyl alcohol (25∶24∶1) and isolated by overnight precipitation at −20°C using 3 M sodium acetate and 20 µg µL^−1^ glycogen. Poly(A) mRNA was purified following manufacturer's instructions using the MicroPoly(A)Purist Kit (Ambion Applied Biosystems, Scoresby, VIC, Australia); concentrations of mRNA were estimated by ultraviolet absorbance and its integrity was confirmed by gel electrophoresis.

### PCR Suppressive Subtractive Hybridization

Subtractive hybridizations of the holobiont ‘Driver’ and ‘Tester’ cDNA fragments were performed following manufacturer's instructions using the PCR-Select cDNA Subtraction Kit (Clontech Laboratories, Inc., CA, USA). After the final round of PCR amplification to enrich for sequences up-regulated in response to enhanced exposure to solar irradiation, a cDNA library was constructed by shotgun cloning the subtracted PCR products into a pCR II-TOPO vector followed by selection for kanamycin-resistant *E. coli* transformants (Invitrogen, Mount Waverley, VIC, Australia). Differential screening was then performed following the manufacturer's protocol to eliminate false positives using the PCR-Select Differential Screening Kit (Clontech Laboratories). Detection followed the Hybond-N+ protocol for dot-blotting nucleic acids using DIG labelled dUTPs (GE Healthcare Bio-Sciences, Rydalmere, NSW, Australia) incorporated into ‘Driver’ and ‘Tester’ cDNAs using PCR primers provided in the screening kit. DNA sequencing (Macrogen Inc., Seoul, South Korea) was obtained for all clones confirmed to contain PCR-SSH sequences.

### Gene annotation and bioinformatics

The cDNA sequences were trimmed to remove vector sequences that may bias the integrity of homology-based searches using the NCBI UniVec database (ftp://ftp.ncbi.nih.gov/pub/UniVec/). The trimmed sequences were then translated into all six reading frames using the EMBL-EBI Transeq server (http://www.ebi.ac.uk/emboss/transeq/). Functional annotation of the insert sequences was performed in a three-step procedure: (i) Similarity searches used the local Smith-Waterman services at EBI (http://www.ebi.ac.uk/Tools/MPsrch/index.html) for initial comparison of amino-acid sequences against all ESTs deposited in GenBank and coral EST libraries in the public domain (http://cnidbase.org/index.cgi); (ii) Annotation of these ESTs using tBLASTx search of the non-redundant nucleotide (nr/nt) database (http://blast.ncbi.nlm.nih.gov/Blast.cgi) and KEGG Orthology (KO) databases (http://www.genome.jp/kegg/ko.html); (iii) Application of the nr/nt and KO results to select the ESTs with significant scores and e-values to assign general biological function according to KEGG BRITE Hierarchical Classification of Protein Families (http://www.genome.jp/kegg/brite.html). Protein sequences for identification of putative MAA biosynthetic genes were extracted from the PROSITE database (http://www.expasy.ch/prosite/) using key words for extracting the sequences described for “methylation”, “dehydrogenation”, “reduction” and “hydroxylation”. Computer programs to facilitate data searching and handling were written using the BioPerl module of Perl (http://www.bioperl.org/wiki/Main_Page).

### PCR amplification of putative MAA biosynthetic sequences from host and algal gDNA from the coral *Acropora millepora*

Coral spermatozoa and cultured dinoflagellate symbionts were used as genetically pure sources of coral animal and dinoflagellate genomic DNA, respectively. Spermatozoa from a colony of a closely related acroprid coral *A. millepora* were collected during the 2005 spawning season from a specimen taken at Magnetic Island, Townsville, Australia. *Symnbiodinium* sp. of clade D1 was isolated from this coral and established in culture. The PCR primer designs for each of the putative MAA pathway encoding genes are as follows: WD008 Forward 5′-GGGGGTAATAATTCTAGCGCGCCTACC-3′, Reverse 5′CCGTCACCAGCAAGGAATA AACAACG-3′, expected product length 164 nt; WD036 Forward 5′-AGGGGCGAGGGGA AAAAGAAGAGTAGAA-3′, Reverse 5′-TCCCTTCCCTCCTCTCTCCCTCTCTATG-3′, expected product length 230 nt; WD103 Forward 5′-CTTCTCACTCTCCCCTCGCCTTAA ACTG-3′, Reverse 5′-GTTAGCGTTGGATAGTGGAAGGGACGAC-3′, expected product length 167 nt. The PCR amplification cycles were the same for each set of primer pairs with a hot start at 94°C for 5 min prior to amplification in 35 repetitive cycles: denaturation at 94°C for 30 sec, annealing at 68°C for 30 sec followed by 30 sec extension at 72°C.

## Supporting Information

Figure S1 Chemical structures of MAAs identified in the coral *Acropora microphthalma*.
(0.14 MB TIF)

Figure S2 Profile of gene expression in *A. microphthalma* exposed to high solar irradiance.
(0.04 MB XLS)

Figure S3 The gel shows PCR amplification of genetically pure DNA templates using primers designed on the sequences of the putative MAA pathway and indicates that these sequences are encoded within the coral and not the algal symbiont.
(0.18 MB PPT)

Figure S4 UV-absorption spectra of methanolic extracts of the coral *Acropora microphthalma* before and after high UV/light exposure.
(0.01 MB PDF)

Table S1 Laboratory codes and corresponding GenBank Accession numbers for the 141 cDNA clones.
(0.03 MB DOC)

Table S2 Amino acid alignments for hypothetical enzymes predicted for MAA biosynthesis.
(0.03 MB DOC)

Table S3 Known cyanobacterial MAA-producers contain a fused aroB (3-dehydroquinate synthase) and *O*-methyltransferase gene, which could theoretically combine the shikimic acid pathway production of 3-dehydroquinate with the first *O*-methylation step of MAA biosynthesis. The data was generated by BLAST searching using as a seed the protein sequence of the fused aroB-OMT protein from the dinoflagellate *Oxyrrhis marina* [25].
(0.06 MB DOC)

Table S4 Comparison of active site residues from 2-*epi*-5-*epi*-valiolone synthase and 3-dehydroquinate synthase enzymes from non-MAA producing prokaryotes and from cyanobacteria known or are likely to produce MAAs. (Alignment is available on request).
(0.10 MB DOC)

Text S1 A discussion supporting vesicular transport and exocytosis as a cellular mechanism of coral bleaching.
(0.08 MB DOC)
