# Supplementary material for: Gene Expression in the Scleractinian Acropora microphthalma Exposed to High Solar Irradiance Reveals Elements of Photoprotection and Coral Bleaching
Source: PLoS One. 2010 Nov 12;5(11):e13975. doi: 10.1371/journal.pone.0013975 (PMC2980464; doi:10.1371/journal.pone.0013975)
Supplement: Figure S3 — The gel shows PCR amplification of genetically pure DNA templates using primers designed on the sequences of the putative MAA pathway and indicates that these sequences are encoded within the coral and not the algal symbiont. (0.18 MB PPT) [file pone.0013975.s003.ppt]

## Slide 1
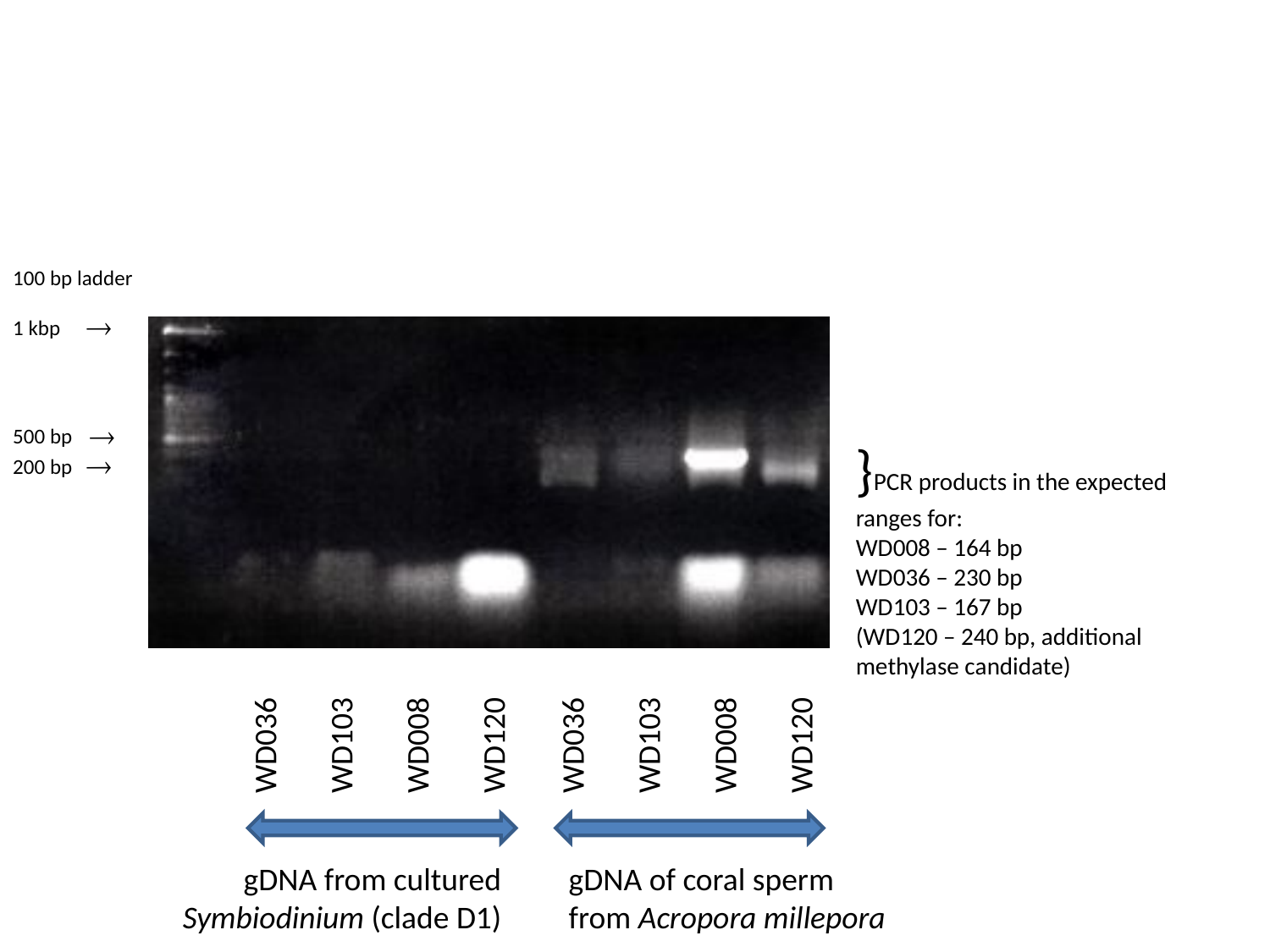

100 bp ladder
1 kbp

500 bp

}PCR products in the expected ranges for:
WD008 – 164 bp
WD036 – 230 bp
WD103 – 167 bp
(WD120 – 240 bp, additional methylase candidate)
200 bp

WD036
WD103
WD008
WD120
WD036
WD103
WD008
WD120
gDNA from cultured
Symbiodinium (clade D1)
gDNA of coral sperm
from Acropora millepora
