# Supplementary figures and images for: Gene Expression in the Scleractinian Acropora microphthalma Exposed to High Solar Irradiance Reveals Elements of Photoprotection and Coral Bleaching
Source: PLoS One. 2010 Nov 12;5(11):e13975. doi: 10.1371/journal.pone.0013975 (PMC2980464; doi:10.1371/journal.pone.0013975)

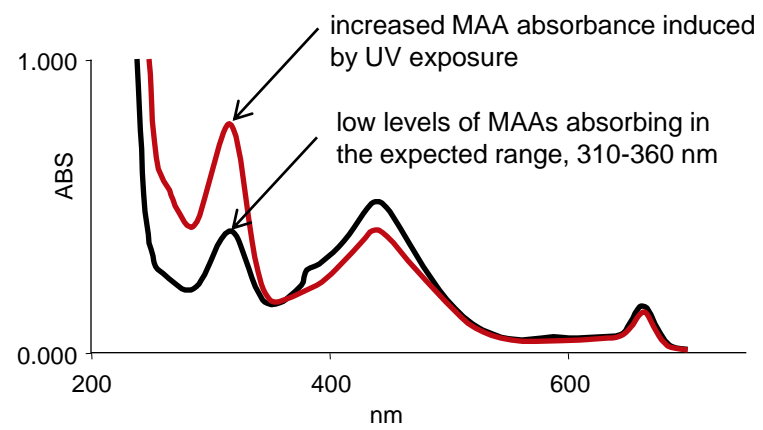

Supplement: Figure S4 — UV-absorption spectra of methanolic extracts of the coral Acropora microphthalma before and after high UV/light exposure. (0.01 MB PDF) [file pone.0013975.s004.pdf]
