# Supplementary material for: Gene Expression in the Scleractinian Acropora microphthalma Exposed to High Solar Irradiance Reveals Elements of Photoprotection and Coral Bleaching
Source: PLoS One. 2010 Nov 12;5(11):e13975. doi: 10.1371/journal.pone.0013975 (PMC2980464; doi:10.1371/journal.pone.0013975)
Supplement: Table S1 — Laboratory codes and corresponding GenBank Accession numbers for the 141 cDNA clones. (0.03 MB DOC) [file pone.0013975.s005.doc]

dbEST_Id User_Id GenBank_Accn

======= ======= ============

65860333 WD001 GR212027

65860334 WD002 GR212028

65860335 WD003 GR212029

65860336 WD007 GR212030

65860337 WD008 GR212031

65860338 WD010 GR212032

65860339 WD011 GR212033

65860340 WD012 GR212034

65860341 WD013 GR212035

65860342 WD016 GR212036

65860343 WD017 GR212037

65860344 WD019 GR212038

65860345 WD020 GR212039

65860346 WD021 GR212040

65860347 WD022 GR212041

65860348 WD025 GR212042

65860349 WD026 GR212043

65860350 WD027 GR212044

65860351 WD028 GR212045

65860352 WD029 GR212046

65860353 WD030 GR212047

65860354 WD031 GR212048

65860355 WD032 GR212049

65860356 WD033 GR212050

65860357 WD034 GR212051

65860358 WD035 GR212052

65860359 WD036 GR212053

65860360 WD037 GR212054

65860361 WD038 GR212055

65860362 WD039 GR212056

65860363 WD040 GR212057

65860364 WD041 GR212058

65860365 WD042 GR212059

65860366 WD043 GR212060

65860367 WD044 GR212061

65860368 WD045 GR212062

65860369 WD046 GR212063

65860370 WD047 GR212064

65860371 WD048 GR212065

65860372 WD049 GR212066

65860373 WD050 GR212067

65860374 WD051 GR212068

65860375 WD052 GR212069

65860376 WD053 GR212070

65860377 WD054 GR212071

65860378 WD055 GR212072

65860379 WD056 GR212073

65860380 WD057 GR212074

65860381 WD058 GR212075

65860382 WD059 GR212076

65860383 WD060 GR212077

65860384 WD061 GR212078

65860385 WD062 GR212079

65860386 WD063 GR212080

65860387 WD065 GR212081

65860388 WD066 GR212082

65860389 WD067 GR212083

65860390 WD068 GR212084

65860391 WD069 GR212085

65860392 WD071 GR212086

65860393 WD072 GR212087

65860394 WD073 GR212088

65860395 WD074 GR212089

65860396 WD075 GR212090

65860397 WD076 GR212091

65860398 WD077 GR212092

65860399 WD078 GR212093

65860400 WD079 GR212094

65860401 WD081 GR212095

65860402 WD084 GR212096

65860403 WD085 GR212097

65860404 WD086 GR212098

65860405 WD087 GR212099

65860406 WD088 GR212100

65860407 WD089 GR212101

65860408 WD090 GR212102

65860409 WD091 GR212103

65860410 WD094 GR212104

65860411 WD095 GR212105

65860412 WD096 GR212106

65860413 WD097 GR212107

65860414 WD098 GR212108

65860415 WD099 GR212109

65860416 WD100 GR212110

65860417 WD101 GR212111

65860418 WD102 GR212112

65860419 WD103 GR212113

65860420 WD104 GR212114

65860421 WD106 GR212115

65860422 WD107 GR212116

65860423 WD109 GR212117

65860424 WD110 GR212118

65860425 WD112 GR212119

65860426 WD113 GR212120

65860427 WD116 GR212121

65860428 WD117 GR212122

65860429 WD119 GR212123

65860430 WD120 GR212124

65860431 WD121 GR212125

65860432 WD122 GR212126

65860433 WD123 GR212127

65860434 WD124 GR212128

65860435 WD125 GR212129

65860436 WD126 GR212130

65860437 WD127 GR212131

65860438 WD128 GR212132

65860439 WD129 GR212133

65860440 WD130 GR212134

65860441 WD131 GR212135

65860442 WD132 GR212136

65860443 WD134 GR212137

65860444 WD135 GR212138

65860445 WD136 GR212139

65860446 WD137 GR212140

65860447 WD138 GR212141

65860448 WD139 GR212142

65860449 WD142 GR212143

65860450 WD143 GR212144

65860451 WD144 GR212145

65860452 WD145 GR212146

65860453 WD146 GR212147

65860454 WD147 GR212148

65860455 WD148 GR212149

65860456 WD149 GR212150

65860457 WD150 GR212151

65860458 WD151 GR212152

65860459 WD152 GR212153

65860460 WD153 GR212154

65860461 WD154 GR212155

65860462 WD155 GR212156

65860463 WD156 GR212157

65860464 WD157 GR212158

65860465 WD158 GR212159

65860466 WD159 GR212160

65860467 WD160 GR212161

65860468 WD163 GR212162

65860469 WD164 GR212163

65860470 WD165 GR212164

65860471 WD009 GR212165

65860472 WD064 GR212166

65860477 WD023 GR212167

65860478 WD083 GR212168
