# Supplementary material for: Gene Expression in the Scleractinian Acropora microphthalma Exposed to High Solar Irradiance Reveals Elements of Photoprotection and Coral Bleaching
Source: PLoS One. 2010 Nov 12;5(11):e13975. doi: 10.1371/journal.pone.0013975 (PMC2980464; doi:10.1371/journal.pone.0013975)
Supplement: Table S2 — Amino acid alignments for hypothetical enzymes predicted for MAA biosynthesis. (0.03 MB DOC) [file pone.0013975.s006.doc]

WD008 – Putative hydroxylase

>gi|undefined gnl|BL_ORD_ID|6347 sp|Q1LX59|C2512_DANRE Cholesterol 25-hydroxylas

e-like protein 1, member 2 OS=Danio rerio GN=ch25hl1.2 PE=3 SV=1

Length: 276 aa

Query frame: +1

Score: 73, Expect: 1.6

Identical: 17/40 (42.5%), Positive: 25/40 (62.5%)

Indels: 2/40 (5%), Gaps: 1

Q: 1672 LSSPLFPHLVILPNYFLLSLYYPICDFL--YFITLYLYKV 1785

| ||||| ++ + +||+| | | || | + +| ||+

D: 35 LRSPLFPVVLTVSSYFVLVLPYLSCDILGRKWPAIYRYKI 74

WD036 – Putative *O*-methyltransferase

>gi|undefined gnl|BL_ORD_ID|467437 sp|Q94AI4|METL1_ARATH Methyltransferase-like protein 1 OS=Arabidopsis thaliana GN=EMB1691 PE=1 SV=1

Length: 775 aa

Query frame: +1

Score: 181, Expect: 4e-11

Identical: 92/417 (22.1%), Positive: 157/417 (37.6%)

Indels: 33/417 (7.91%), Gaps: 11

Q: 34 GRRGARGKRRVESAAGEETKKKGGREERKGESSTTG-GREGVDENEERKG-ERTEGRGQR 207

| | + + ++++ | + + || + ++ | + | || | ||| |

D: 25 GDRSEKRRMSLKASDFESSSRSGGSKSKEDNKSVVDVEHQDRDSKRERDGRERTHG---- 80

Q: 208 TGGRTKKRREKKHREGERREGRETKRRGAGERRRGGREKRKIGGRKARKRGRRDGRNQRE 387

+ + ||+ | +| + + | | |+ + | || ++ |

D: 81 SSSDSSKRKRWDEAGGLVNDGDHKSSKLSDSRHDSGGERVSVSNEHGESR--RDLKSDRS 138

Q: 388 RKEKRRRRRGRRAG-----RGGRGKREEGKTPSGGKKRKGREGTGRRKKGGESKGEEREE 552

| | + + | || |+ || | + || || + +

D: 139 LKTSSRDEKSKSRGVKDDDRGSPLKKTSGKDGSEVVREVGRSN--------RSKTPDADY 190

Q: 553 EEKGGKREKERGEGNGQERGEGERDKGRRREGRKKRRRAREKGRGGEKKRKAGG--KERR 726

|++ |+ || | | +| |+ | +|| | + | |+| +| | +|

D: 191 EKEKYSRKDERSRG----RDDGWSDRDRDQEGLKDNWKRRHSS-SGDKDQKDGDLLYDRG 245

Q: 727 EKRKSAKERRDQGGEKGKREAREGRRRRGKKGRRKEEEGERGEREQRGREEKRERRKRRE 906

+|+ ++ |++ | + | | |+ | +| + | + + + +

D: 246 REREFPRQGRERS-EGERSHGRLGGRKDGNRGEAVKALSSGGVSNENYDVIEIQTKPHDY 304

Q: 907 QRGNREERGRKRKRR*GDPKKEESGREEGERQGKKGRTNRRERRRKERGEENDRERGGAE 1086

|| + | |+ | || ++|| ||++ | | |

D: 305 VRGESGPNFARMTESGQQPPKKPSNNEEEWAHNQEGRQRSETFGFGSYGEDSRDEAGEAS 364

Q: 1087 GGDSRRRERRRKRRREGD----ERMRRGGKREWKRGGRRRRGGGKGKNAEREREHEG 1245

| + | ++ | + || + | | ||||+ | +|

D: 365 SDYSGAKARNQRGSTPGRTNFVQTPNRGYQTPQGTRGNRPLRGGKGRPAGGRENQQG 421

WD036 – Putative dehydrogenase

>gi|undefined gnl|BL_ORD_ID|708224 tr|Q3JSI4|Q3JSI4_BURP1 Oxidoreductase, short chain dehydrogenase/reductase family OS=Burkholderia pseudomallei(strain 1710b) GN=BURPS1710b_2074 PE=3 SV=1

Length: 759 aa

Query frame: +1

Score: 255, Expect: 8e-20

Identical: 123/432 (28.5%), Positive: 162/432 (37.5%)

Indels: 35/432 (8.1%), Gaps: 11

Q: 37 RRGARGKRRVESAAGEETKKKGGREERKGESSTTGGREGVDENEERKGERTEGRGQRTGG 216

||||| | ||| + +|| | + || | +

D: 50 RRGARMHR-----AGERRRARGGHRRRVDRGAL---REQPVRRLGHSGRPRDAAAASVPH 101

Q: 217 RTKKRREKKHREGERREGRETKRRGAGERRRGGREKRKIGGRKARKRGRRDGRNQRERKE 396

|+++ | | | | | | |+||| + ||+ || | |

D: 102 LDGPARDRRGRLGARARRRRTAWRDHGQRRRQLLRLHE-DAVAAREAPRRVGERALLRAA 160

Q: 397 KRRRR---RGRRAGRGGRGKREEGKTPSGGKKRKGREGTGRRKKGGESKGEEREEEEKGG 567

+| || || || || + + || | | | |+ +||

D: 161 ERVRRVSADGRARGRSQRGGQHRRRLRSG-PPRLVRRGARHAGHSGDDDAGAARRFGRGG 219

Q: 568 KREKERGEGNGQERGEGERDKGRRREGRKKRRR---AREKGRGGEKKRKAGGKERREKRK 738

| | + || | +|||| ||+ | | | ++ | |+ |+

D: 220 GRAAARMGARARPRGGHARRRGRRRRGRRDVRGGCDAARPARRDDRHEHVLGLPCRDGRR 279

Q: 739 SAKERRDQGGEKGKREAREGRRRRGKKGRRKEEEGERGEREQRGREEKRERRKRREQRGN 918

+|+ | | + +| || | || | | | + | | ||+|

D: 280 AARAR--QHAARVRRPARPVRVRRRDHGGRVGIVVSRAVLPRGNRGRARGRRRRASPARG 337

Q: 919 REERGRKRKRR*GDPKKEESGREEGERQGKKGRTNRRERRRKERGEENDRERGGAEGGDS 1098

| ||++ +|| | + + | | || | | |

D: 338 RRRRGQRGQRRRAVPAVPDGRAQPG--------VGREGERRVRRPVAISHARASVPRGAR 389

Q: 1099 RRRERRRKRRREGDERMRRGGKREWKRGGRRRRGGGKGKNAEREREH------EGRGRGG 1260

| | + + | | | || | | ||||| | +|| | | + || |

D: 390 RARVCAQAQHRGG--RGRRARARCAARRGRRRRAFGS-VDAEHRRHHRLPGVDDRRGGRG 446

Q: 1261 EEKRGRKRRKRK 1296

+ || || |+

D: 447 GDGRGAARRARR 458

WD103 – Putative reductase

>gi|undefined gnl|BL_ORD_ID|631733 tr|C4B488|C4B488_BURMA Short-chain dehydrogenase/reductase SDR (Fragment) OS=Burkholderia mallei GB8 horse 4 GN=BMAGB8_3535 PE=4 SV=1

Length: 191 aa

Query frame: -2

Score: 87, Expect: 1.1

Identical: 47/162 (29%), Positive: 61/162 (37.7%)

Indels: 15/162 (9.26%), Gaps: 5

Q: 897 RRGDRGDRPKRNGGRTRREKRKGQAES*RSLQTRGEAGRRTPKAERCNRMEVRRYGRRGG 718

|| || || | | | | | |+ + | |+ +| | | | |

D: 8 RRRSRGARPPRGGARRRSGGRSGRRAARRRLRRGARPAGLRDRAGRARR------GHRAR 61

Q: 717 GEAQIKDRAQQGSWMRG*ARLDAESTSDVLSARAWGPFEDMTLWRGREEENARARRQTKR 538

++ | + |+ | || | + || | |||+ |||

D: 62 RRLRVA-RGRDGAQRRRAARARAHARRRDARRRA----RRRTRARGRDSS---ARRNAVS 113

Q: 537 CLSMVAVP-GRSGSSNSRSGATESSLLLTRKGERGGRRRRAA 415

+ || |+| + || | + | || |||

D: 114 SGARAAVAFARAGRAASRDGGAGARARAEGARRRARRRARAA 155

WD137 – Putative *O*-methyltransferase

>gi|undefined gnl|BL_ORD_ID|492759 tr|Q8I555|Q8I555_PLAF7 Protein-S-isoprenylcysteine O-methyltransferase, putative OS=Plasmodium falciparum (isolate 3D7) GN=PFL1780w PE=4 SV=2

Length: 509 aa

Query frame: +1

Score: 114, Expect: 6e-04

Identical: 39/139 (28.1%), Positive: 63/139 (45.3%)

Indels: 39/139 (28.1%), Gaps: 5

Q: 166 FYFALLLQI*IYLSNYFLFLSLSSKLDFFFNFPLLLLFLCIILFYYIFIYILLNPI*NVM 345

+ || || + ||+| + ++ | |||| | +| || |+ ++

D: 80 YIFAKLLLL------YFIFFFIHFVINLFNNFPLNLFYLIIISFHLSEFFL--------- 124

Q: 346 CFSHNIILSSLFHFL--PSSYFSIFFLIQYSTYYIIILLNILYILFFFYTFILLYSIY-- 513

| || ++ ++|| |+| + ||++ ||+ | ||+ |+ +| |

D: 125 SFLHNKENANYYNFLVNPNSVYVYFFILTLFEYYLKI---------FFFVFLNVYQKYIN 175

Q: 514 -----------SFFFLLNY 537

++||| ||

D: 176 NQKILHKVLLINYFFLRNY 194

WD036 – Putative dehydrogenase

>gi|undefined gnl|BL_ORD_ID|186603 tr|C7TQP1|C7TQP1_9BILA NADH dehydrogenase subunit 2 (Fragment) OS=Radopholus similis GN=nad2 PE=4 SV=1

Length: 275 aa

Query frame: +1

Score: 124, Expect: 2e-05

Identical: 44/167 (26.3%), Positive: 79/167 (47.3%)

Indels: 25/167 (15%), Gaps: 4

Q: 103 IPWIKPSSTIFIPMYFPL*IFFYFALLLQI*IYLSNYFLFLSLSSKLDFF--FNFPLLLL 276

| | | | | + ++ + | | |++| + || | |+|| ||+ ||++

D: 21 IIWWLSFFLINIIMLLLVKLYNVYLLYFIIYIFISEFLGFLFL---LNFFNYFNYILLMI 77

Q: 277 FLCIILFYYIFIYILLNPI*NVMCFS---HNIILSSLFHFLPSSYFSIFFLIQYSTYYII 447

+ | |+| |+|| | |++ + ++ +|++| +| ++ |+|+

D: 78 KVGISPFFYWLIFILFNLKSNILLYYLVYMKLVYLPVFYYLYLNYLWMYLFGLVIIYFIM 137

Q: 448 ILLNILYILFFFYT-----------------FILLYSIYSFFFLLNY 537

| + ++||| | |+|++ | |||++ |

D: 138 IFFKLNSLMFFFSTQESFLLLLVMLSFSLMDFLLMFFYYFFFFMMIY 184
