# Supplementary material for: Gene Expression in the Scleractinian Acropora microphthalma Exposed to High Solar Irradiance Reveals Elements of Photoprotection and Coral Bleaching
Source: PLoS One. 2010 Nov 12;5(11):e13975. doi: 10.1371/journal.pone.0013975 (PMC2980464; doi:10.1371/journal.pone.0013975)
Supplement: Table S3 — Known cyanobacterial MAA-producers contain a fused aroB (3-dehydroquinate synthase) and O-methyltransferase gene, which could theoretically combine the shikimic acid pathway production of 3-dehydroquinate with the first O-methylation step of MAA biosynthesis. The data was generated by BLAST searching using as a seed the protein sequence of the fused aroB-OMT protein from the dinoflagellate Oxyrrhis marina [25]. (0.06 MB DOC) [file pone.0013975.s007.doc]

Synechococcus sp. WH 8102 Nucleotide accession [NC_005070](http://www.ncbi.nlm.nih.gov/entrez/viewer.fcgi?val=NC_005070)

Region: 1305255..1306370

>gi|33865844|ref|NP_897403.1| 3-dehydroquinate synthase

MVSSPTLRRIAVALERNPYEVVIGAGGLSRLGDELLKAGIQPGRRILVVSNPDVAAPYGEGCLQSLRQQG

FHADLLVIDAGEERKTPATVAAIHDSAFEQRLERSSLMLALGGGVVGDMTGFAAATWLRGIQVIQVPTTL

LAMVDAAIGGKTGVNHPGGKNLIGAFHQPSLVLIDPETLNTLPEREFRAGMAEVIKYGILGDPALFQCLE

EGPEPNSAAGLGNSRLETILERSAAAKARVVAADEREGGLRAVLNYGHTFGHVVETLCGYGTWLHGEAVA

IGMVAVGQLAVNRGSWTADEAGRQTQLIQRCGLPTAWPDLDPDAVLRTLQGDKKVKDGRLRFVLPMAIGR

VEIRDDISRDEILHCLDQLRG

>gi|33865845|ref|NP_897404.1| hypothetical protein SYNW1311 MRCVPMDLLQWRAFSPMIPTVADCPAWLATLLHQAGGTVPFRQFMDWALHHPEHGYYGSGRVRIGPQGDF

ATSPSLGPDFATLLGRQLIDLLRNLSDQASTLSLVEVGPGEGDLAADLLTVLARQAPDLIERCELVLVER

SPSLRQRQQQRLEGISGCPVRWCGIEELQSSPIQGVLLAHELLDAFPVDRLVLKQGELALQGVRLQQNDQ

LTSVPLALPDTLQEQLQTSGLELPPPGSEDGWTTEWHSNLRPWFGTLASAVSDGALLVIDYAHEASRYYT

ARRSEGTLMAYRDGMAGMNPLAHAGEQDLTAHLCIETLTQAAAHHGWQLRDQRRQGEALLALGLANDLHA

LQQLPASELAEALRRREALLRLVDPAALGDFRWLLFSRGAGADCFSLATGPDNAGFHPG

Synechococcus sp. RCC307 Nucleotide accession [NC_009482](http://www.ncbi.nlm.nih.gov/entrez/viewer.fcgi?val=NC_009482)

Region: 1258515..1259636

>gi|148242518|ref|YP_001227675.1| 3-dehydroquinate synthase MDSGGAMTTVTVELTRPYDVVIGQGSLHQLGERLKQQAIRAGTKILVVSNPVVAEHYGATVMGSLSSAGY

EAELLVIEAGEEQKTPATVAQIHDKAYAMQLERSSLMLALGGGVVGDMTGFAAATWLRGVAVVQVPTTLL

AMVDAAIGGKTGVNHPGGKNLIGAFHQPRLVMIDSATLGTLPIREVHAGMAEVIKYGVIGDPVLFEALEA

HGPLDSLEAIGSTLMQQILRRSAAAKARVVAHDEREGSLRAILNYGHTLGHVVETLTGYGTYLHGEAVGI

GMHAAGLLSQRLGLWSADEQQRQLTLLKRCGLPWQAPELNTEAIRTAILRDKKVQNGRVRFVLPTAIGQV

VIRDDVDIEAAVAALKATSGVEA

>gi|148242519|ref|YP_001227676.1| SAM-dependent methyltransferase MPNPCCPPDHRDAIDERYGAAALEQEVCLCAAVPFDPALLAAIPKEVVSRDYGCGNPTRWVRPGDRVLDL

GSGSGKNAFICSQLVGAEGSVLGLDRNPNMLELANSACGSVASQIGYGNVSFRRAEIDQLAEDLDGQPLL

ADGCIDVVLSNCVLNLVQPSRRGQLLQEIRRVTAPGGRLAISDIICDKPVPLELQQDPDLWSGCISGAWL

ESEFCAAFEQLGFSKVALVDRQEEPWQVHAGIAFRSATLTAELPCC

Synechococcus sp. CC9902 Nucleotide accession [NC_007513](http://www.ncbi.nlm.nih.gov/entrez/viewer.fcgi?val=NC_007513)

Region: 1014682..1015815

>gi|78184623|ref|YP_377058.1| 3-dehydroquinate synthase MGSPSSMTTTIHAHRINVALERNPYDIVIGDGVLGIVGDELHRLGAKPGKKILVVSNADVAGPYGEACLN

SLKEKGFNVELLVIEAGEEQKHLRTVSQIHDAAFAFKLERSSMLLALGGGVVGDMTGFAAATWLRGVGVV

QVPTTLLAMVDASIGGKTGVNHPGGKNLIGAFHQPHLVLIDPTTLDTLPIREFRAGMAEVIKYGILGDTD

LFLELESCKDPSSPNGLGRTTLESILQRSAAAKASIVAADEREGGLRAVLNYGHTFGHVVEALCGYGTWL

HGEAVALGMVAVGELAVQRGNWSRGDAERQKQLIAKAGLPTTWPELNLEAVLHTLQGDKKVRDGQLRFVI

PTEIGSVEIQNDISRDEITHCLEHLAT

>gi|78184622|ref|YP_377057.1| hypothetical protein Syncc9902_1049 MESMSAACPSWLAMHLKQLGGVTSFRQYMDLALNDPNHGFYGSGRAQISRDGDFVTSTALGTDFAGLLAT

QVERWLAELPADLPTLSLIEIGPGEGDLLADLVDALTDLSPQILHRLELVLVEANPGMKQRQQARLQHLT

NIPMRWCSLDELLAAPLRGLVLAHELLDALPVDRLTFDDGVMWQQLVELDDDGALVFSKGHVPPQLAAEI

ERVCKRCELVLPPPDAEPGWTTEWHSGSSNWFKQLSQALDQGVLLVVDYALEMHRYYSARRSDGTLMAVQ

AQRAGLSPLDKPGSQDLTAHICIETVEDAAVQAGWTCMGQLRQGEALLALGLAERLYGLQSLPPGDLPQA

LQRREAMLRLVDPSGLGDFRWLLFGKGVNPASFKLPSAPDSA

Synechococcus sp. WH 7803 Nucleotide accession [NC_009481](http://www.ncbi.nlm.nih.gov/entrez/viewer.fcgi?val=NC_009481)

Region: 1096885..1097988

>gi|148239540|ref|YP_001224927.1| 3-dehydroquinate synthase MEPESRIRVALERNPYDVIIGSGQLARVGDALSALQVRAGTRILVVSNPDVADHYAAQCLSSLEAAGLKP

VLLTIDAGEEQKTLDTLRIILDAAQHNGLERTSLMLALGGGVVGDMTGFAAACWLRGIRVIQVPTTLLAM

VDASIGGKTGVNHPCGKNLIGAFHQPSLVMIDPLTLRTLPAREFRAGMAEVIKYGVIGDPELFERLEQAE

DLSDPGRMTPELLQTILERSAQAKAAVVAADEREGGRRAILNYGHTFGHVVETLTGYGTWLHGEAVAIGM

ATVGQLAVNKGLWTDSDQQRQLTLIQKAGLPTAWPSLNIDKVLDTLRSDKKVRHGQLRFVLPDAIGSVLI

SDQIRDDDVRQCLTSQH

>gi|148239539|ref|YP_001224926.1| hypothetical protein SynWH7803_1203 MVSDVCCPAWLLDRLRQSGGEIPFSMFMDWALHDPVHGAYGAGHLTVGPDGDFATSPSLGEDFADLLVDQ

LVDWLGDLGERHPDDRLSVVDVGPGEGTLTAQLIPLLRRKAPALAERLDCVLVECNPGMESRQKQRLGAS

PAIPCRWTSLEDLRRNPLIGVVVAHELLDALPVERLVLRAGTLHRQMVRLRVEGASAQIHLAEGSFEGEL

RAQFQEDCARSGMVIPPAGAEDGWTTEWHASVSPWMRDAAAAVRQGVLLVVDYAYEADRYYTRHRSDGTL

LAYREQVATHDVLRNAGTQDITAHLCVEAVVEAAERNGWMHEGQRRQGEALLALGLAERFTALQSLPAEQ

LSEALQRRETLLRLVDPACLGDLRWMVFHRQSERREDDPWTRSRLLHDPPALSAGSSDTA

Synechococcus sp. CC9311 Nucleotide accession [NC_008319](http://www.ncbi.nlm.nih.gov/entrez/viewer.fcgi?val=NC_008319)

Region: 1306482..1307615

>gi|113954762|ref|YP_730635.1| 3-dehydroquinate synthase MIQSQPSTTASAELRRLSVALERNPYDVVITAGGIDHLGTELLRLGIREHTKILVVSNADVATHYGSRCL

NSLEQAGFQSTLLTIPAGEEQKTLNTFSTILDAAKEKGLERQSLMLALGGGIVGDMTGFAAACWLRGIGV

VQVPTTLLAMVDAAIGGKTGVNHPSGKNLIGAFHQPRLVMIDPDTLQTLPAREFRAGLAEVIKYGVIGDS

DLFELLERSVSFDSPLSISKELLATMLERSAQAKADVVAADEKEGGQRAILNYGHTFGHVVETLTGYGTW

LHGEAVAIGMVAVAALAVQRGVMAQSDAERQTRLIKSAGLPSQWPDLNPDLVLKTLQGDKKVRNGRLRFV

LPSSIGVVSIVDDVTHEEIRACLASMR

>gi|113953923|ref|YP_730636.1| hypothetical protein sync_1431 MAWALHDPEHGAYGSGQLKIGKGGDFVTSATLGPDFSALLGCQLVQWVRTLALNYPTETLSIVEVGPGEG

ELSCDLIDHLAEHLPDLMHRLELVLVETNPGMEQRQRNRLKQHQVSQQAQPLFPQRWTSLSDLKAKPVIG

VLIAHELLDAFPVERLELIDGQLRRQTVQFQQEHVGGGDLHWGTEPIPQSLQERMNATLSATQIALPPPD

AEDGWTTEWHDACASWFAEASEALIAGHLLVVDYVLEAHRYYSARRREGTLMAYRNQRASSSVLVDAGQQ

DLTAHLCLETMVHQATTNGWSLEGQCRQGEALLALGLAERFSSLQQLPGSQLAEVLQRREALLRLVDPAC

LGEFRWLSFLRFNPLVPRNGTGERSQFLQEPTGINALTPSML

Synechococcus sp. CC9605 Nucleotide accession [NC_007516](http://www.ncbi.nlm.nih.gov/entrez/viewer.fcgi?val=NC_007516)

Region: 1360583..1361698

>gi|78212980|ref|YP_381759.1| 3-dehydroquinate synthase MTTITPLHHIRVALERNPYEVVIGNGGLARLGQQMLDAGVQADRRVLVVSNPDVANPYGDACLNSLREAG

FSVELLVIDAGEHQKTPATVAEIHDAAYSAKLERSSLMVALGGGVVGDMTGFAAATWLRGIQVVQVPTTL

LAMVDASIGGKTGVNHPRGKNLIGAFHQPRLVLIDPSTLNTLPEREFRAGMAEVIKYGILGDTALFEELE

ACPDPSTPAGLGAERLSSILQRSAAAKARVVAADEKEGSLRAILNYGHTFGHVVETLCGYGTWLHGEAVA

IGMVAVGELAVLRGSWSRDDAERQRRLIESAGLPTAWPDLSADAVLNSLQGDKKVRDGRLRFVMPTGIGS

VEIRDDVSREEILSCLERLKG

>gi|78212981|ref|YP_381760.1| hypothetical protein Syncc9605_1451 MELLQRPAFSPMEPMVASCPEWLATHLHQAGGAVPFSRFMDLALNEPEHGYYGSGRARIGAQGDFVTSPA

LGSDFAVLLAPQILAWLTSIPRSDPDQRLSIVEIGPGEGHLARDLVAALHGADPELLARIELVLVEANPG

MRRRQQALLQEADDLPLRWCSLDELRRAPVQGVVIAHELLDALPVERLIWREGSLQQQWVELAPKGDLRT

THRPLPDGLHQEIRRVCGQSGIQLPPPDAEEGWTTEWNSAMLDWFAAAAAAVDAGVLLVIDYALEAERYY

TARRSDGTLMAVCAQQAGLSPLDQPGEQDLTAHLCIEVVDEAAQRNGWLVGDQIKQGEALLALGLAQRLH

GLQQLPGQQLAEALQRREALLRLVDPAGLGAFRWLTYLRGLPEGGFSLSGAPGSSEFPHD

Synechococcus sp. RS9916 Nucleotide accession [NZ_AAUA01000002](http://www.ncbi.nlm.nih.gov/entrez/viewer.fcgi?val=NZ_AAUA01000002)

Region: 248000..249094

>gi|116074787|ref|ZP_01472048.1| 3-dehydroquinate synthase MQRITVKLASNPYDVVIGSGGLQNLGQEMLNSGISAPRKVLVVSNADVAGPYGATVLESLRSSGFEADSL

VIEAGEDQKHLGTVSQIHDAAHRLRLERGSLMVALGGGVVGDMTGFAAATWLRGIAVVQVPTTLLAMVDA

AIGGKTGVNHPGGKNLIGAFHQPKLVLIDPDTLNTLPDREFRAGMAEVIKYGVIGDPGLFDLLESCDGLS

EASALPDGVLQTILERSAQAKANVVAADEREGGQRAILNYGHTFGHVVENLTGYGTWLHGEAVAIGMVAV

GELAVQRGHWSRDQANRQRRLIQKCGLPTVWPPLDPSAVLETLQGDKKVQDGTVRFVVPDAIGAVSIRSD

VNTDEVKACLAALS

>gi|116074786|ref|ZP_01472047.1| hypothetical protein RS9916_29669 MDRLGAPCPAWMVTRFEAEGGRMSFRRFMELALHDPVDGAYGSGRLRVGTKGDFVTSPSMGSDFAALLAT

QLAEWLDQIHAEAPASPLSLVEVGPGEGDLAADVWAELHRLNPAWIGQLELVLVERNPGMESRQRERLAA

SAPGQVRWTTLDKLAADPIRGVLVAHELLDAFPVERLIWRDGAMRQMGVVLTSDDAGQKVLHWDDQMLPD

SIQQQLDWADRHCGISVPPAQVPEGWTTEWHGEVAPWLEQVAKAVASGVMLIVDYAHDAERYYSARRFAG

TLLAYHQQQASDELLADAGCRDLTAHLCIDTLLAQARDQGWTVLGQCRQGEALLALGLGERLHNLQRLPA

TELPQALQRREALLRLVDPAGLGEFRWIALSRFPDEPRESELISRCLEAPKGFS

Synechococcus sp. BL107 Nucleotide accession [NZ_AATZ01000001](http://www.ncbi.nlm.nih.gov/entrez/viewer.fcgi?val=NZ_AATZ01000001)

Region: 71775..72887

>gi|116070488|ref|ZP_01467757.1| 3-dehydroquinate synthase MTTTNAHRINVALERNPYDIVIGDGVLGTVGEELSRLGAKPGKKILVVSNADVAVPYGDACLNSLREKGY

NVEMLVIEAGEEQKLLRTVSQIHDAAFSFKLERSSMMLALGGGVVGDMTGFASATWLRGVGVVQVPTTLL

AMVDASIGGKTGVNHPGGKNLIGAFHQPNLVLIDPTTLDTLPIREFRAGMAEVIKYGILGDPDLFNELES

CDDPSSPGGLGRTRLESILRRSASAKARIVAADEREGGLRAVLNYGHTFGHVVEALCGYGTWLHGEAVAL

GMVAVGELAVQRGNWSRIDAERQKRLIAKAGLPTTWPELNLEAVLQTLQGDKKVRDGQLRFVIPTGIGSV

EIQNDISRDEITHCLEQLAA

>gi|116070487|ref|ZP_01467756.1| hypothetical protein BL107_12615 MHLKQLGGVTSFRRYMDLALNDPNDGFYGSGRARVSRDGDFVTSPALGSDFAGLLASQVVRWLAELPADL

PTLSLIEIGPGEGDLLADLVDAIADQSPQMLHRLELVLVEANPGMKQRQQERLQHQTKFPMRWCGLDELV

AAPLRGVVLAHELLDALPVERLTYDEGVMWQQLVELDDDGALVFSKGPLPTQLADEIERVCKRCGLDLPP

PDADPGWTTEWHSDSLGWFTQLGQVLDQGVLLVVDYALEMHRYYSARRSDGTLMAVQAQRAGLSPLHKPG

SQDLTAHICIETVEDAAVQAGWSCLGQLRQGEALLALGLAERLYGLQSLPPGDLPQALQRREAMLRLVDP

SGLGDFRWLLFGKRVNPASFKLPTAPDSA

Synechococcus sp. WH 7805 Nucleotide accession [NZ_AAOK01000003](http://www.ncbi.nlm.nih.gov/entrez/viewer.fcgi?val=NZ_AAOK01000003)

Region: 292140..293273

>gi|88808613|ref|ZP_01124123.1| 3-dehydroquinate synthase MTITPETTTAMEPQARIRVALERNPYDVIIGRGQLNHVGDAISALQVREGTRILVVSNPDVADHYAAQCL

TSLETGGFRPVLLQIDAGEEQKTLDTFRIILDAAQRNGLERTSLMLALGGGVVGDMTGFAAACWLRGIRV

VQVPTTLLAMVDASIGGKTGVNHACGKNLIGAFHQPSLVLIDPLTLRTLPTREFRAGMAEVIKYGVIGDP

ELFDRLEQADDLSEPGRMDQELLQMILERSAQAKAAVVAADEREGGRRAILNYGHTFGHVVETLTRYGTW

LHGEAVAIGMAAVGDLAVIKGLWTETDQQRQLTLIRKAGLPTQWPSLAIDRVLDTLRSDKKVRHGQLRFV

VPNAIGSVLISDQISDDDVRQCLEGQH

>gi|88808614|ref|ZP_01124124.1| hypothetical protein WH7805_02952 MVSDVCCPAWLLDRLRQSGGEVPFSLFMHWALHDPDHGAYGSGRLAVGPEGDFTTSPSLGEDFAELLVDQ

LVDWLQALAEFHPDDRLSVVDVGPGEGTLTAQLIPLLLSKAPGLVDRLDCVLVECNPGMELRQKQRLGAS

PAIPCRWSSLEDLRLNPLVGVVVAHELLDALSVERLVLRSGTLQRQMVRLRDEGSSAQIHLAEGPFDGEL

RARFQSECDRSGMVIPPVGAEDGWTTEWHASVAPWMRDAAAAVKQGVLLVVDYAFEADRYYTCHRSDGTL

LAYQQQVATNDVLRNAGTQDITAHLCVDGVVAAAEMHGWMFEGHRRQGEALLALGLAERFSALQSLPAAQ

LGEALRRRETLLRLVDPSCLGDLRWMVFHRQNERPENALGQLSRLLRDPQQVSAGPLDTA

Cyanothece sp. CCY0110

Nucleotide accession [NZ_AAXW01000005](http://www.ncbi.nlm.nih.gov/entrez/viewer.fcgi?val=NZ_AAXW01000005) Region: 65082..66404

>gi|126656689|ref|ZP_01727903.1| 3-dehydroquinate synthase MVQAKTLTEKTISKSNQIIQFNHPVKYRTSEWYTGHGEIVASGDNRSFEVSASYNLKATVKLVNEILNPD

NPTLAEIYSRRGRCVAIVDLTVDELYGEAIRYYFEHHEINLEIMPCRAWESDKTPQTVNNLLAFLGKDGC

DVSRNEPVLVIGGGVLSDVAGLACALQHRRTPYIMIGTTVVAAIDAGPSPRTCTNGSQFKNSMGVYHPPV

LTLVDRTFFRTLDTGHIRNGMAEIIKMAVTDDPVLFELMEEYGSRLLETHFATLEGDEELAEIADEVIYR

ALFSYMKHEGTNMFETYQDRPHAYGHTWSPRFEPAAKLMHGHAVSIGMAFGASLATEMGWLSTEDRDRII

ALCSSIGLSVFHPILEDMDIMLEGQKNMRRKRGEGGLWAPLPTGIGECDFAQEVSSGLLQQAVDKHKTLC

VSLPSGGEGKEMYLSDLGLA

>gi|126656688|ref|ZP_01727902.1| O-methyltransferase

Nucleotide accession [NZ_AAXW01000005](http://www.ncbi.nlm.nih.gov/entrez/viewer.fcgi?val=NZ_AAXW01000005) Region: 64236..65069

MSQLLTTPPTARPVTPLGILVEKLENVRKLAENEQVSASLLTALQEVEALAGGIDPYLDDCTTAESAALA

NLAQKTAQEDWSKQFSDGATVRQLEQEMLSGHIEGQTLKLFVSMMGAKRILEVGMFTGYSALAMAEALPE

DGCVIACEVDQYVADFARACFNESPHGNKIDIKVAPALETMKALADAKECFDLVFIDADKTEYIDYFKLL

LERNLVASGAVICVDNTLLQGQPYLPSQQRTANGEAIAQFNRFVADDDRVEQVLLPLRDGLTLIRRK

Crocosphaera watsonii WH 8501 Nucleotide accession [NZ_AADV02000002](http://www.ncbi.nlm.nih.gov/entrez/viewer.fcgi?val=NZ_AADV02000002)

Region: 683581..684903

>gi|67921329|ref|ZP_00514848.1| 3-dehydroquinate synthase MVQAKPLNDKTASESNQIIQFNHPVKYRTSEWYTGHGEIVASGDNRSFEVSASYNLKATVKLVNDILSPE

NPTLAEIYSSLGRCVAIVDLTIDELYGEAIRKYFEYHEISLELYPCRAWESDKTPATVNKILSYLGKDGS

DVSRNEPVLVMGGGVLSDVAGLACALQHRRTPYVMIGTTVIAAIDAGPSPRTCTNGSQFKNSMGVYHPPV

LTLVDRTFFRTLETGHVRHGMSEIIKMAVTDDPVLFELMEQYGARLVETHFATLGGDAELSEIADEVIYR

ALFSYMKHEGTNMFETYQDRPHAYGHTWSPRFEPAAKLMHGHAVSIGMAFGASLATEMGWLSAEDRDRII

SLCTSMGLSVYHSILEDMDIMLEGQKNMRRKRGEGGLWAPLPTTIGECDYAQEVSSGLLEKAVQHHKEIC

VSLPSGGEGKEMYLSDLGLA

>gi|67921328|ref|ZP_00514847.1| Caffeoyl-CoA O-methyltransferase MCFLTIWGRRQGNVSQRSRFSIGGLTMSQLLTTPPVARPVTPLGILVEKLENVRKLAENEEVSASLLNAL

QEVEALAGGLDPYLDETTTEESAALANLAQKTAQEDWSKRFSDGETVRQLEQEMLSGHIEGQTLKIFVSM

MGAKRILEVGMFTGYSALAMAEALPEDGCIIACEVDQYVADFARSCFDESPHGSKIDIKVAPALETMQGL

AETQECFDLVFIDADKGGYIDYFKLLLESNLLAPGGIICVDNTLLQGQPYLPEQQRTANGEAIAQFNRFV

ADDDRVEQVLLPLRDGLTLIRRK

Prochlorococcus marinus subsp. pastoris str. CCMP1986 Nucleotide accession [NC_005072](http://www.ncbi.nlm.nih.gov/entrez/viewer.fcgi?val=NC_005072) Region: 650190..651281

>gi|33861239|ref|NP_892800.1| 3-dehydroquinate synthase MHKNKILVPLSNNSYEVIIKQGLINNIGEELIRIGINSNRKILIVSNKEISTLFGRKLLNNLKKNNFNAE

IFNIKAGESHKNFASLSEIFNAAFEVGLDRNSLLIALGGGIVGDVTGFAAATWLRGIEYIQIPTTLLSMV

DSSVGGKTAVNHPKGKNLIGAFYQPKAVFIDPETLITLPTREFKAGMAEVIKYGVIKDKSLFEYLENEKN

RDKILNLENESLIKIINKSIKTKACIVSEDEKENGIRAILNYGHSFGHVIENLCGYGEYLHGEAISIGMK

IAGDIATEKNLWSKEHSLRQDHLIESYGLPIQTPKIKKNDVMKILMGDKKVRNGKMRFILPIELGEVDIF

NDINESQFLKYFN

>gi|33861238|ref|NP_892799.1| hypothetical protein PMM0681 MESLPANNQEWLIKKIIKKGGTISFYDYMDIVLNDLNNGYYGSGKANLGSKGDFVTSPSMSDDFAFLLSK

QIYEWLIQVKSKSNCDDKLSVIEFGAGDGSLMSGLLEYFFINDKKILKNVCFIIIEPNKGMIKKQQKKLE

KYLKLGFDILWRCLEDLEDRSLNGVVLANEVLDALPVERIINLKGKMQRQGVSIDKKSGRLFFEAISITK

ELEKSIASAQEKLDINIPPKYAPEGWTTEWHIDNKKWLMAIYAKINNGILLIIDYAKEAKRYYSLGNNNG

TLISYKNQKIVENIFESPGDCDLTSHVCIESLIYDSETLGFETIGIVKQGEALLSLGLAERLFEIQNELK

DDISKALSRREALLRLVDPICLGDFKWFVFSKFNNKKFKINSLCIR

Prochlorococcus marinus str. NATL2A Nucleotide accession [NC_007335](http://www.ncbi.nlm.nih.gov/entrez/viewer.fcgi?val=NC_007335)

Region: 670935..672041

>gi|72381956|ref|YP_291311.1| 3-dehydroquinate synthase MNKDNHHIKVSLTNNPYEIVIGKSSLESIGDELFNIGFREGLKVLVVSNKEVSDHYGDCIIKSLIKSKFK

PKLLIIKAGEDQKNQSSIDLIHNAAYEARLERGSLMIALGGGVIGDMTGFAAATWLRGVNVVQIPTTLLA

MVDASIGGKTGINHSKGKNLIGAFHQPRLVLIDPKTLITLPSREFKAGMAEIIKYGVISDLELFELLERQ

ENISDLSNIKEKLLIEIIKRSAKSKAEIVIKDEKESGVRAFLNYGHTFGHVIENLCGYGKWLHGEAVAMG

MVAVGQLAVQRGLWKEDNAKRQKRLIEKAGLPSNWPQLEIESVLSSLQGDKKVKNGKVSFVMPLKIGDVK

LFNNISNKEIRECLQKIS

>gi|72381955|ref|YP_291310.1| hypothetical protein PMN2A_0115

MIDPTARCPKWLIDRIGDSGGSISFYRYMDLVLNDPDNGVYSTGKLNIGKNGDFCTSPSLSNDFARLLAI

QVVDWLLDLEKSGIDSKLLSLVEIGPGEGTLSRDLIVAIAEIAPALICKVELVLVELNVGMRRRQEKVVN

NLEGINCRWSSIEDLILRPVTGVVIANEVLDAFPVERLVFNDNKVFRQGVSLKKINDEYSLEFVDLKPTS

KIIKFLKESKSLLKIEFPPKDICNRWVTEWHCDVPSWFGNLSKVLIDGALLVVDYAMESKRYYNAMRQDG

TLISYRNHVANPNVLKDAGLCDLTTHLCIESTINYALFNGWKFMGETRQGQALLALGLSTFLYSLQNNIN

NDLSAALNRRESLLRLVDPIGLGDFRWLAFQKDNSDDLILGKRFLEEPIS

Prochlorococcus marinus str. MIT 9215 Nucleotide accession [NC_009840](http://www.ncbi.nlm.nih.gov/entrez/viewer.fcgi?val=NC_009840)

Region: 685632..686723

>gi|157413102|ref|YP_001483968.1| 3-dehydroquinate synthase MNKRKILVPLGEKSYEVTLEAGILNNISEELLKIGITKKRKILVISNEEISNLYGKKFLNNLKDNKFQAK

MFLIKAGESYKNLKTLSEIYDVAFEFGLDRNSIIIALGGGIVGDVSGFAAATWLRGIEYIQIPTTLLSMV

DSSVGGKTGVNHPKGKNLIGAFNQPKAVFIDPETLKSLPKREFSAGMAEVIKYGVIRDKELFEYLEIDKN

KNELINLKNEYLIKIINSSIKTKSHVVSQDEHENGVRAILNYGHSFGHVIENLCGYGKFLHGEAISIGMN

IAGKIAIEKGLWSKEELERQRVLLESYDLPTEIPKINKEDVLTILMGDKKVRDGKMRFILPKEIGAVDIY

DDVEDSLFLKFFS

>gi|157413101|ref|YP_001483967.1| hypothetical protein P9215_07661 MNSLPANNPDWLVKKIIKMGGTISFYDFMNFALNDPINGYYGSGKAELGVRGDFVTSPSLSDDFAFLVGK

QIEDWLIQFKSSFLSNQTLSITEFGAGDGSFMSGLIKYFLKKSKNFLEGVSFVIIEPNEGMVEKQKNKLE

EFLNLGIDILWKGLDEVEENNINGIVLANEVLDALPVERITFLKGKLIRQAVSIDKKSNKLFFDKMPITL

ELEKSFELAKSELGITIPPEDALEGWTTEWHVDNSKWLEAIYGKINNGILLIIDYAKEAKKYYNSKNSDG

TIVSYENQKMKNNVLDSPGNCDLTSHVCIETLINDAESLGFNTDGITKQGEALLALGLAERLYGIQKEFK

ENLSNALLRREALLRLVDPVCLGDFKWFVFKKFNEKKMNIKSTCLR

Prochlorococcus marinus str. MIT 9301 Nucleotide accession [NC_009091](http://www.ncbi.nlm.nih.gov/entrez/viewer.fcgi?val=NC_009091)

Region: 655091..656182

>gi|126696073|ref|YP_001090959.1| 3-dehydroquinate synthase MNKRKILVPLGDKSYEVTLEAGILNNISEELLKIGITKNRKILVISNEEISNLYGEKFLNNLKDNKFQAK

MVLIKAGESYKNLKTLSEIYDVAFEFGLDRNSIIIALGGGIVGDVSGFAAATWLRGIEYIQIPTTLLSMV

DSSVGGKTGVNHPKGKNLIGAFNQPKAVFIDPETLKSLPKREFSAGMAEVIKYGVIRDKELFKYLEIEKN

KNELINLKNEYLIKIINSSIKTKSHIVSQDEHENGVRAILNYGHSFGHVIENLCGYGKFLHGEAISIGMN

IAGKIAIEKGLWSKEELERQKILLESYDLPTEIPKINKEDVLTILMGDKKVRDGKMRFILPKEIGAVDIY

DDVEDSLFLKFFS

>gi|126696072|ref|YP_001090958.1| hypothetical protein P9301_07341 MNSLPANNPDWLVKKIIKMGGTISFYDFMNFALNDPINGYYGSGKAELGVRGDFVTSPALSDDFAFLVGK

QIEDWLIQFKNSFLSNQKLAVIEFGAGDGSFMSGLIKYFLENNKNFLEGVSFVIIEPNEGMVEKQKNKLE

EFLNLGIDILWKGLDEVEENNINGIVLANEVLDALPVERITFAKGKLIRQAVSIDKKSHKLFFDKMPITR

ELEKSFELAKSELGITIPPADALEGWTTEWHVDNSKWLEAIYGKINNGILLIIDYAKEAKKYYNSKNSDG

TIVSYENQKMKNNVLDSPGNCDLTSHVCIETLINDAENLGFNTDGITKQGEALLALGLAERLYGIQKEFK

EDLSNALLRREALLRLVDPVCLGDFKWFVFKKFNEKKMNINSTCLR

Prochlorococcus marinus str. MIT 9303 Nucleotide accession [NC_008820](http://www.ncbi.nlm.nih.gov/entrez/viewer.fcgi?val=NC_008820)

Region: 1356858..1357976

>gi|124023259|ref|YP_001017566.1| 3-dehydroquinate synthase MSAFVNIDAQRIPVALSHQPYEVVIGGEGLRGVGKELRRAGLKEGIKVLVVSNADVAEPYGDLCLQSLSD

SGYRPTLLVIEAGEDQKTPVSVALIHDAAYEAKLERGSLMVALGGGVVGDMTGFAAATWLRGISVVQLPT

TLLAMVDAAIGGKTGVNHPGGKNLIGAFHQPRLVLIDPSTLNTLPEREFRAGMAEVIKYGVIGDAALFQL

LEGIPELNTPSQLHADLLEKILERSALAKSRVVSSDEREGGLRAILNYGHTFGHVVETLCGYGTWLHGEA

VAIGMVAVGELAVLRQSWNRDDANRQKSLIAKAGLPIAWPKLDPEEVLYTLQGDKKVKDGKLRFVIPTGI

GNVEIKNDVSREEIRKCLSELS

>gi|124023260|ref|YP_001017567.1| hypothetical protein P9303_15581 MDLCSVPCPAWLANRIVQAGGSISFHQYMDWALHDQVYGAYASGQLHIGRQGDFATSPSLGADFAQLLAI

QLADWFQQLQQHVDKGRSLSLIEVGPGEGDLSADLISALEDLCPALIPRLELVLVESNKAMVQRQRERLK

SVTTVPIHWRSLDELAQAPAIGVMLAHEMLDALPVERLVWRDQRLWRQGVCLENVDSVAHLRFTELSLTD

ALHSALTEARMFWGIQIPPPDADDGWCSEWHGELKSWLSQAASALLYGPLLIIDYALEARRYYSAMRPCG

TLMAYRQQRASGALLQDPGRWDLTAHLCLETLQHQAEQQGWSFLGESRQGQALLALGLAEKLHALQSLPT

SQLSAALNRREALLRLVDPAGLGEFRWLAFELRPKPSVEAKVGELRCRFLEEPVS

Prochlorococcus marinus str. NATL1A Nucleotide accession [NC_008819](http://www.ncbi.nlm.nih.gov/entrez/viewer.fcgi?val=NC_008819)

Region: 683270..684376

>gi|124025449|ref|YP_001014565.1| 3-dehydroquinate synthase MNKDNHHIKVSLTNNPYEIVIGKNSLESIGDELFNIGFREGLKVLVVSNKEVSDHYGDCIIKSLIKSKFK

PKLLIIKAGEDQKNQSSIDLIHNAAYEARLERGSLMIALGGGVIGDMTGFAAATWLRGVNVVQIPTTLLA

MVDASIGGKTGINHSKGKNLIGAFHQPRLVLIDPKTLISLPSREFKAGMAEIIKYGVISDLELFDLLERQ

ENIADLSNIKEKLLLEIIKRSAKSKAEIVIKDEKESGVRAFLNYGHTFGHVIENLCGYGKWLHGEAVAMG

MVAVGQLAVQRGLWNEDNAKRQKRLIEKAGLPSNWPKLDIESVLSSLQGDKKVKNGKVSFVMPLKIGDVK

LFNNISNKEIRECLQKIS

>gi|124025448|ref|YP_001014564.1| hypothetical protein NATL1_07411 MIDPIARCPKWLIDRIGDSGGSISFYRYMDLVLNDPDNGFYSTGKLNIGKNGDFCTSPSLSNDFARLLAI

QVVDWLLDLEKSGIDSKLLSLIEIGPGEGTLSRDLILAIAEIAPALICKIELVLVELNVGMRRRQEKVVN

NLEGINCRWSSIEDLILRPVNGVVIANEVLDAFPVERLVFSDNKVFRQGVGLKKINDENYLEFVDLKPTS

KIIKFLKESNSLLKIEFPPKDICNRWVTEWHCDVPSWFGNLSKVLIDGALLVVDYAMESKRYYNAMRQEG

TLISYRNHVANPNVLKDAGLCDLTAHLCIESTINYALFNGWKFMGETRQGQALLALGLSNFLYSLQNNSN

NDLSAALNRRESLLRLVDPIGLGDFRWLAFQKDNSDDLILRNRFLEEPIS

Prochlorococcus marinus str. MIT 9515 Nucleotide accession [NC_008817](http://www.ncbi.nlm.nih.gov/entrez/viewer.fcgi?val=NC_008817)

Region: 684434..685525

>gi|123965990|ref|YP_001011071.1| 3-dehydroquinate synthase MNKNKIIVPLSNNSYEVTIRQGIINSIGKELTQIGINNNRKILIVSNKEISNLFGSKLLNDLKKYNFSAE

IFNIKAGESYKNLASLREIYDAAFEFGLDRNALLIALGGGIVGDVTGFAAATWLRGIDYIQIPTTLLSMV

DSSVGGKTAVNHPKGKNLIGAFYQPKAVFIDPETLKTLPIREFKAGMAEVIKYGVIKDKELFEYLEIDKN

REKILNLDNESLIKIINKSIRTKSYIVSKDEKENGIRAILNYGHSFGHVIENLCGYGEYLHGEAISIGMK

IAGDISTEKNLWLKEDSLRQDKLIESYGLPTQTPKIKKHDVITILMGDKKVRDGKMRFILPKGIGEVDIF

NDIKESQFLKYFD

>gi|123965989|ref|YP_001011070.1| hypothetical protein P9515_07541

MNSLPANNPEWLIKKIIKKGGTISFYDYMNLVLNDPNNGYYGSGKANLGSKGDFVTAPSMSDDFAFFLSK

QIYQWLIQVKSKSVSFDNLSVLEFGAGDGSLMSGLLHYLFIYNKQFFSNVSFIIIEPNKGMINKQKEKLE

KYLNLGFNIMWRSLEELEDKSLNGVILANEVLDALPVERLINLKGKIYRQGVSLDKETGRLFFKEIKISK

ELEKSIVFAKENLNIYIPPKDAPEGWTTEWHTDNKSWLKAVYEKINNGILLVIDYAKEAKRYYSLSNNNG

TLISYKNQKIIEDVFESPGNCDLTSHICIESLIYDSETLGFETMGIVKQGEALLLLGLAERLFEIQNELK

DDISKALSRREALLRLVDPICLGDFKWFVFSKFKNKKIKIKSRCIS

Prochlorococcus marinus str. AS9601 Nucleotide Accession [NC_008816](http://www.ncbi.nlm.nih.gov/entrez/viewer.fcgi?val=NC_008816)

Region: 656255..657346

>gi|123968272|ref|YP_001009130.1| 3-dehydroquinate synthase MNKRKILVPLGDKSYEVTLEAGILNNISEELLKIGITKKRKILVISNEEISNLYGEKFLNNLKDNKFQAK

MFLIKAGESYKNLKTLSEIYDVAFEFGLDRNSIIIALGGGIVGDVSGFAAATWLRGIEYIQIPTTLLSMV

DSSVGGKTGVNHPKGKNLIGAFNQPKAVFIDPETLKSLPKREFSAGMAEVIKYGVIRDKELFEYLEIEKN

KNELINLKNEYLIKIINSSIKTKSNVVSQDEHENGVRAILNYGHSFGHVIENLCGYGKFLHGEAISIGMN

IAGKIAIEKGLWSKEELERQRILLESYDLPTEIPKINKEDVLTILMGDKKVRDGKMRFILPKEIGAVDIY

DDVEDSLFLKFFS

>gi|123968271|ref|YP_001009129.1| hypothetical protein A9601_07361 MNSLPANNPDWLVKKIIKMGGTISFYDFMNFALNDPINGYYGSGKAELGVRGDFVTSPSLSDDFAFLVGK

QIEDWLIQFKSSFLSNETLSVTEFGAGDGSFMSGLIKYFLENSKNFLEGISFVIIEPNEGMVEKQKNKLE

EFLNLGIDILWKGLDEVEENNINGIVLANEVLDALPVERITFSKGKLIRQAVSIDKKSHKLFFDKMPITR

ELEKSFELAKSELGITIPPEDALEGWTTEWHVDNSKWLEAIYGKINNGILLIIDYAKEAKKYYNSKNSDG

TIVSYENQKMRNNVLDSPGNCDLTSHVCIETLINDAETLGFDTVGITKQGEALLALGLAERLYGIQKEFK

ENLSNALLRREALLRLVDPVCLGDFKWFVFKKFNEKKININSTCLR

Prochlorococcus marinus str. MIT 9312 Nucleotide accession [NC_007577](http://www.ncbi.nlm.nih.gov/entrez/viewer.fcgi?val=NC_007577)

Region: 643794..644885

>gi|78779067|ref|YP_397179.1| 3-dehydroquinate synthase MNKRKILVPLGNKSYEVTIEAGILNNISEELLKIGITKNRKILVISNEEISNFYGEKFLNDLKDNKFQVQ

MFLIKAGESYKNLKTLSEIYDVAFEFGLDRNSIIIALGGGIVGDVSGFAAATWLRGIEYIQIPTTLLSMV

DSSVGGKTGVNHPKGKNLIGAFNQPKAVFIDPETLKSLPKREFSAGMAEVIKYGVIRDKELFEYLEIEKN

KNELINLKNEYLIKIINSSIKTKSYIVSQDEHENGVRAILNYGHSFGHVIENLCGYGKFLHGEAISIGMN

IAGEIAIDKGLWSKEELERQKNLLKSYDLPTEIPKINKEDVLTILMGDKKVRNGKMRFILPKEIGVVDIY

DDVEDALFLKFFS

>gi|78779066|ref|YP_397178.1| hypothetical protein PMT9312_0681

MNSLPANNPDWLVKKIIKMGGTISFYDFMNFVLNDPINGYYGSGKAVLGVRGDFVTSTSLSDDFAFLAGK

QIEDWLIQFKSSFLSNQKLAVIEFGAGDGSFMSGLIKYFLENNKNFLEGVSFLIIEPNKGMVEKQKNKLE

EFLNLGIDILWKGLEEVEENNINGIVLANEVLDALPVERITFSKGKLLRQAVSIDKKSHNLFFDEMPITN

ELDKSIELAKSELGITIPPEDALEGWTTEWHIDNSKWLKAIYGKINNGILLIIDYAKEAKKYYTSKNSDG

TIVSYENQKMTNNVLDSPGNCDLTSHVCIETLIHDAETLGFNTVGITKQGEALLALGLAERLYGIQKEFK

EDLSNALLRREALLRLVDPVCLGDFKWFVFKKFNEKKMNINSTCLR

Lyngbya sp. PCC 8106 Nucleotide accession [AAVU01000005](http://www.ncbi.nlm.nih.gov/entrez/viewer.fcgi?val=AAVU01000005)

Region: 4155..5372

>gi|119485307|ref|ZP_01619635.1| 3-dehydroquinate synthase

MTFTLSKPASKPASQSSSTEYASLKQEVKVTFNYDVHFTRDLFNPDNPMLANVLLSKGEGVKKVIAFVDS

GLPQQTEILAQIQAYSNCHSYALNLKPGSTLIAGGEAAKNNPQLVENLQKIISEAELCRHSYVIAVGGGA

MLDLVGYAAATAHRGIRLIRVPTTVLGQCDSGVGVKNGINAFGKKNFLGTFAPPTLVLNDANFLTTLDDR

NWRSGLAEAVKVALIKDAAFFDFIVNHTEALVNRDLDTMQQIVYRCAKLHLDHIAGGDPFEMGSSRPLDF

GHWAAHRIEYLSNYRLYHGEAVAIGICLDSTYSYLSGLITKQELNEILNLFKALGFRLYDAELSSHFTEP

EHSRSLFRGLSEFQEHLGGELTLMLLATVGQGLEVHQVDLDKYRLAIMMLKEADL

>gi|119485306|ref|ZP_01619634.1| hypothetical protein L8106_09201

MQLTENSTHLTYCTNIHPGESWLEVFDNLKQYIPALKSRLNSEYLGIGLRLSHQATTQLKPSEMSEFKAW

LAKHNLYVFTLNGFPYGGFHNQVVKDQVYAPDWTTSERTNYTQHLAYILAELLPQGVNGSISTLPLSYKP

WFNSSSDINEVFKQSTINLVKVVVELVNLHQKTGKFIHVDLEPEPNGLIENSAEVIDFFENWLLPVGGKQ

LADTLGIDLNTAEKLLRQHIQICYDTCHFSVVYEKPSQVFEQFKQAGILVGKIQISAAIKNQLPQDQSER

DHLAEYLHKFVDSTYLHQVVAAHPDGTFQSYIDLPEALQELRETTAQEWRTHFHIPIFNKTYGVLQSTQD

DILTVLDLLKDYPCEHLEIETYTFQVLPENLKTDLLTSIQREYEWVLQHYQDHSNLYSNQQQTVSSASS
