# Supplementary material for: Gene Expression in the Scleractinian Acropora microphthalma Exposed to High Solar Irradiance Reveals Elements of Photoprotection and Coral Bleaching
Source: PLoS One. 2010 Nov 12;5(11):e13975. doi: 10.1371/journal.pone.0013975 (PMC2980464; doi:10.1371/journal.pone.0013975)
Supplement: Table S4 — Comparison of active site residues from 2-epi-5-epi-valiolone synthase and 3-dehydroquinate synthase enzymes from non-MAA producing prokaryotes and from cyanobacteria known or are likely to produce MAAs. (Alignment is available on request). (0.10 MB DOC) [file pone.0013975.s008.doc]

| Organism | Protein | 182 | 198 | 204 | 214 | 246 | 249 | 302 | 317 | 321 | 324 | 308 | 321 | 388 | Function |
| --- | --- | --- | --- | --- | --- | --- | --- | --- | --- | --- | --- | --- | --- | --- | --- |
| *Actinoplanes* sp. strain 50/110 | **^1^acbC** | R | D | K | N | E | K | ^5^M | ^5^E | ^5^D | H | ^5^P | H | ^5^V | **2-*epi*-5-*epi*-valiolone synthase** |
| *Streptomyces hygroscopicus* subsp. *Jinggangensis* | **^1^valA** | R | D | K | N | E | K | ^5^M | ^5^R | ^5^D | H | ^5^P | H | ^5^V | **2-*epi*-5-*epi*-valiolone synthase** |
| *Actinomyces* sp. Lu 9419 | **^1^cetA** | R | D | K | N | E | K | ^5^M | ^5^E | ^5^D | H | ^5^P | H | ^5^T | **2-*epi*-5-*epi*-valiolone synthase** |
| *Nonomuraea spiralis* | **^1^prlA** | R | D | K | N | E | K | ^5^M | ^5^T | ^5^D | H | ^5^P | H | ^5^T | **2-*epi*-5-*epi*-valiolone synthase** |
| *Aspergillus nidulans* | **^1^aroMA** | R | D | K | N | E | K | K | R | N | H | H | H | K | 3-dehydroquinate synthase |
| *Mycobacterium tuberculosis* | aroB MTCY159.18 | R | D | K | N | E | K | K | R | N | H | H | H | K | 3-dehydroquinate synthase |
| *Escherichia coli* strain K12 | **aroB** JW3352 | R | D | K | N | E | K | K | R | N | H | H | H | K | 3-dehydroquinate synthase |
| *Staphylococcus aureus* strain MRSA252 | **aroB** SAR1476 | R | D | K | N | E | K | K | R | N | H | H | H | K | 3-dehydroquinate synthase |
| *Synechococcus* sp. WH 8102 | ^2^NP_897403.1 | R | D | K | N | E | K | K | R | N | H | H | H | K | 3-dehydroquinate synthase |
| *Synechococcus* sp. RCC307 | ^2^YP_001227675.1 | R | D | K | N | E | K | K | R | N | H | H | H | K | 3-dehydroquinate synthase |
| *Synechococcus* sp. CC9902 | ^2^YP_377058.1 | R | D | K | N | E | K | K | R | N | H | H | H | K | 3-dehydroquinate synthase |
| *Synechococcus* sp. WH 7803 | ^2^YP_001224927.1 | R | D | K | N | E | K | K | R | N | H | H | H | K | 3-dehydroquinate synthase |
| *Synechococcus* sp. CC9311 | ^2^YP_730635.1 | R | D | K | N | E | K | K | R | N | H | H | H | K | 3-dehydroquinate synthase |
| *Synechococcus* sp. CC9605 | ^2^YP_381759.1 | R | D | K | N | E | K | K | R | N | H | H | H | K | 3-dehydroquinate synthase |
| *Synechococcus* sp. RS9916 | ^2^ZP_01472048.1 | R | D | K | N | E | K | K | R | N | H | H | H | K | 3-dehydroquinate synthase |
| *Synechococcus* sp. BL107 | ^2^ZP_01467757.1 | R | D | K | N | E | K | K | R | N | H | H | H | K | 3-dehydroquinate synthase |
| *Synechococcus* sp. WH 7805 | ^2^ZP_01124123.1 | R | D | K | N | E | K | K | R | N | H | H | H | K | 3-dehydroquinate synthase |
| *Cyanothece* sp. CCY0110 | ^2^ZP_01727903.1 | R | D | ^4^R | N | E | K | ^5^H | ^5^D | ^5^A | H | ^5^P | H | ^4^R | **2-*epi*-5-*epi*-valiolone synthase fused to *O*-methyltransferase??** |
| *Crocosphaera watsonii* WH 8501 | ^2^ZP_00514848.1 | R | D | ^4^R | N | E | K | ^5^H | ^5^D | ^5^A | H | ^5^P | H | ^4^R | **2-*epi*-5-*epi*-valiolone synthase fused to *O*-methyltransferase??** |
| *Prochlorococcus marinus* subsp. pastoris str. CCMP1986 | ^2^NP_892800.1 | R | D | K | N | E | K | K | R | N | H | H | H | K | 3-dehydroquinate synthase |
| *Prochlorococcus marinus* str. NATL2A | ^2^YP_291311.1 | R | D | K | N | E | K | K | R | N | H | H | H | K | 3-dehydroquinate synthase |
| *Prochlorococcus marinus* str. MIT 9215N | ^2^YP_001483968.1 | R | D | K | N | E | K | K | R | N | H | H | H | K | 3-dehydroquinate synthase |
| *Prochlorococcus marinus* str. MIT 9301 | ^2^YP_001090959.1 | R | D | K | N | E | K | K | R | N | H | H | H | K | 3-dehydroquinate synthase |
| *Prochlorococcus marinus* str. MIT 9303 | ^2^YP_001017566.1 | R | D | K | N | E | K | K | R | N | H | H | H | K | 3-dehydroquinate synthase |
| *Prochlorococcus marinus* str. NATL1A | ^2^YP_001014565.1 | R | D | K | N | E | K | K | R | N | H | H | H | K | 3-dehydroquinate synthase |
| *Prochlorococcus* marinus str. MIT 9515 | ^2^YP_001011071.1 | R | D | K | N | E | K | K | R | N | H | H | H | K | 3-dehydroquinate synthase |
| *Prochlorococcus marinus* str. AS9601 | ^2^YP_001009130.1 | R | D | K | N | E | K | K | R | N | H | H | H | K | 3-dehydroquinate synthase |
| *Prochlorococcus marinus* str. MIT 9312 | ^2^YP_397179.1 | R | D | K | N | E | K | K | R | N | H | H | H | K | 3-dehydroquinate synthase |
| *Lyngbya* sp. PCC 8106 | ^2^ZP_01619635.1 | R | D | K | N | E | K | ^5^H | ^5^S | ^5^D | H | H | H | ^4^Q | **2-*epi*-5-*epi*-valiolone synthase fused to *O*-methyltransferase??** |
| Anabaena variabilis ATCC 29413 | ^3^YP_324358.1 | R | D | K | N | E | K | ^5^M | ^5^D | ^5^A | H | ^5^P | H | ^5^L | **2-*epi*-5-*epi*-valiolone synthase** |

Active site residues based upon Carpenter *et al*., (1998) *Nature* 394: 299-302 and Stratmann *et al*., (1999) *J Biol Chem*. 274:10889-10896.

^1^Used as an example of either **2-*epi*-5-*epi*-valiolone synthase** or 3-dehydroquinate synthase in the alignment of Wu *et al*., (2007) C*hemBioChem* 8:239 – 248.

^2^Cyanobacterial strains with a putative fused 3-dehydroquinate synthase *O*-methyltransferase gene. See Table S3 for details.

^3^Identified originally as 3-dehydroquinate synthase by Singh and Hader (2010) [*Genomics*.](javascript:AL_get(this, 'jour', 'Genomics.');) 95:120-128, but latter as **2-*epi*-5-*epi*-valiolone synthase by Balskus and Walsh (2010) *Science*** Sep 2. [Epub ahead of print].

^4^Conservative and ^5^non-conservativer substitutions from the 3-dehydroquinate synthase peptide crystal structure of *Aspergillus nidulans* pentafunctional aroM complex. See Carpenter *et al*., (1998) *Nature* 394: 299-302 and Stratmann *et al*., (1999) *J Biol Chem*. 274:10889-10896.
