# Supplementary material for: Gene Expression in the Scleractinian Acropora microphthalma Exposed to High Solar Irradiance Reveals Elements of Photoprotection and Coral Bleaching
Source: PLoS One. 2010 Nov 12;5(11):e13975. doi: 10.1371/journal.pone.0013975 (PMC2980464; doi:10.1371/journal.pone.0013975)
Supplement: Text S1 — A discussion supporting vesicular transport and exocytosis as a cellular mechanism of coral bleaching. (0.08 MB DOC) [file pone.0013975.s009.doc]

**(a) Evidence for exocytosis in coral bleaching**

In the mutualistic partnership of cnidarian-algal symbiosis, the algae reside and proliferate within a specialised phagosome (the symbiosome) inside the host gastrodermal cells. The intracellular persistence of the alga-containing symbiosome relies on the exclusion or retention of small Rab GTPase proteins. Significantly, symbiosomes of the sea anemone *Aiptasia pulchella* enveloping healthy dinoflagellates contain the vesicle-associated membrane protein (VAMP) ApRab5 (Chen *et al*., 2004), a checkpoint antagonist of downstream ApRab7 and ApRab11 proteins that are involved in phagosomal maturation (Chen *et al*., 2003; 2005). Thus, cellular communication and the cross-talk between Rab proteins provide coordination of the endocytic pathway necessary for organelle homeostasis, which in symbiotic corals has long been recognised as essential for the host’s regulation of its endosymbiotic partners to maintain the stability of the symbiosis (Muscatine & Pool, 1979). While Rab GTPases and their effector proteins coordinate consecutive stages of exo- and endocytic vesicular transport (Collins, 2003; Deneka et al., 2003), soluble N-ethylmalemide–sensitive factor attachment receptor (SNARE) proteins are essential for Rab assembly in late-stage docking and the fusion of vesicle membranes necessary to complete endosomal exocytosis (Vassilieva & Nusrat, 2008). Rab proteins impart specificity to SNARE-dependent exocytosis by allowing fusion partners (including the Ca^2+^-sensor synaptotagmin) to pair by distinct binding of vesicle-associated Rab and SNARE proteins prior to membrane fusion (Ohya *et al.*, 2009). In the final step of vesicle exocytosis, a cellular influx of calcium binds to synaptotagmin and actuates the completion of membrane SNARE protein assembly, forming the conducting channel for trans-membrane vesicular transport (Jena, 2009). Coral genes encoding syntaxin-like SNARE proteins have been assigned unambiguously (http://bioinformatics.mpibpc.mpg.de/snare/snareQueryPage. jsp) from coral EST database libraries constructed from expressed mRNA of the post-settlement stage of *A. millepora*, from mRNA of the unfertilized eggs of *Acropora palmata* and from mRNA of the early larval stage of *Monastraea faveolata* (Kloepper *et al*., 2007).

Experiments based on existing models of exocytosis can now be applied to corals to examine the possible role of Rab-GTPase in coordination of vesicular transport, tethering and SNARE membrane fusion (He & Guo, 2009), and to test the hypothesis that vesicular switching of the symbiosome back to a phagosome, followed by expulsion of the endosymbionts by host phagosomal exocytosis, is a realistic cellular model for coral bleaching. From the foregoing evidence emerges an integrative hypothesis for light-induced coral bleaching that would entail physiological cues released by resident algae at the symbiosome membrane affecting alteration of VAMP Rab protein composition to activate the exocytic pathway of SNARE protein assembly in which the final step of symbiont exocytosis is actuated by a stress-induced release of cellular calcium, binding to SNARE synaptotagmin-syntaxin complexes. This mechanism is supported by histological examination asserting that coral host cells are not significantly degraded during bleaching (Strychar & Sammarco, 2009), that intact dinoflagellates *in hospite* are released by exocytosis from the endoderm into the coelenteron cavity in partially bleached corals (Brown *et al*., 1995) and that symbionts freshly expelled during thermal bleaching may even appear healthy and photosynthetically competent (Brown *et al*., 1995; Bhagooli & Hidaka 2004). Consistent also is the observed cytosolic influx of calcium that is recognised as an effector of cnidarian bleaching (Fang *et al*., 1997; Sawyer & Muscatine, 2001), thus providing the synaptotagmin-SNARE protein trigger to complete exocytosis. Compelling evidence for the involvement of SNARE proteins in exocytosis of endosymbiotic dinoflagellates is provided by the observation that treating the coral *Acropora grandis* with N-ethylmaleimide (a specific antagonist of SNARE protein assembly), or with N-(6-aminohexyl)-5-chloro-1-naphthalene-sulfonamide (a specific antagonsist of calmodulin Ca^2+^-regulation), independently inhibited the release of these algae under controlled conditions of coral bleaching (Fang *et al*., 1998; Huang *et al*., 1998).

**(b) Hypothetical trafficking in cnidarian symbioses**

It is tempting to speculate that endocytosis of free-living dinoflagellates in the aposymbiotic life stage of juvenile corals that do not inherit their endosymbionts maternally, the reacquisition of algal partners by bleached specimens and exocytosis of algae during coral bleaching may follow the same reversible exocytic/endocytic membrane trafficking pathways as a complementary mechanism for the establishment of coral symbiosis, in host regulation of symbiont populations and for the release of algae in coral bleaching. Such modulation of symbiont trafficking would allow the host cnidarian to remodel the genetic structure of its endosymbiont populations (“symbiont shuffling”) in response to changing environmental conditions (Berkelmans & van Oppen, 2006; Jones *et al*., 2008). Nonetheless, such processes of symbiont trafficking do not preclude more drastic cytological changes that may occur under severe stress leading to catastrophic coral bleaching and mortality.

**References**

Berkelmans, R. & van Oppen, M. J. H. 2006 The role of zooxanthellae in the thermal tolerance of corals: ‘a nugget of hope’ for coral reefs in an era of climate change. *Proc. R. Soc. B* **273**, 2305-2312. (doi: 10.1098/rspb.2006.3567)

Brown, B. E., Le Tissier, M. D. A. & Bythell, J. C. 1995 Mechanisms of bleaching deduced from histological studies of reef corals sampled during a natural bleaching event. *Mar. Biol.* **122**, 655-663. (doi: 10.1007/BF00350687)

Bhagooli, R. & Hidaka, M. 2004 Release of zooxanthellae with intact photosynthetic activity by the coral *Galaxea fascicularis* in response to high temperature stress. *Mar. Biol*. **145**, 329-337. (doi: 10.1007/s00227-004-1309-7)

Chen, M.-C., Cheng, Y.-M., Hong, M.-C. & Fang, L.-S. 2004 Molecular cloning of Rab5 (ApRab5) in *Aiptasia pulchella* and its retention in phagosomes harbouring live zooxanthellae. *Biochem. Biophys. Res. Commun.* **324**, 1024-1033. ([doi:10.1016/j.bbrc.2004.09.151](http://dx.doi.org/10.1016/j.bbrc.2004.09.151))

Chen, M-C., Cheng, Y-M., Hong, M-C., Huang, Y-S., Liu M-C., Cheng Y-M. & Fang, L-S. 2005 ApRab11, a cnidarian homologue of the recycling regulatory protein Rab11, is involved in the establishment and maintenance of the *Aiptasia-Symbiodinium* endosymbiosis. *Biochem. Biophys. Res. Commun.* **338**, 1607-1616.

Chen, M.-C., Cheng, Y.-M., Sung, P.-J., Kuo, C.-E. & Fang, L.-S. 2003 Molecular identification of Rab7 (ApRab7) in *Aiptasia pulchella* and its exclusion from phagosomes harboring zooxanthellae. *Biochem. Biophys. Res. Commun.* **308**, 586-595.

([doi:10.1016/S0006-291X(03)01428-1](http://dx.doi.org/10.1016/S0006-291X(03)01428-1))

Collins, R. N. 2003 Rab and ARF GTPase regulation of exocytosis. *Mol. Membr. Biol.* **20**, 105-115. (**doi:** 10.1080/0968768031000085892)

Deneka, M., Neeft M. & van der Sluijs, P. 2003 Regulation of membrane transport by Rab GTPases. *Crit. Rev. Biochem. Mol. Biol.* **38**, 121-142. (**doi:** 10.1080/713609214)

Fang, L-S., Huang, S-P., & Lin, K-U. 1997 High temperature induces the synthesis of heat-shock proteins and elevation of intracellular calcium in the coral *Acropora grandis*. *Coral Reefs* **16**, 127-131. (doi: 10.1007/s003380050066)

Fang, L-S., Wang, J-T. & Lin, K-L. 1998 The subcellular mechanism of the release of zooxanthellae during coral bleaching. *Proc. Natl. Sci. Counc. R.O.C. (B)* **22**, 150-158.

He, B. & Guo, W. 2009 The exocyst complex in polarized exocytosis. *Curr. Opin. Cell Biol.* **21**, 537-542. (doi: 10.1016/j.ceb.2009.04.007)

Huang, S-P., Lin, K-L., & Fang, L-S. 1998 The involvement of calcium in the process of heat induced coral bleaching. *Zool. Stud.* **37**, 89-94.

Jena, B. P. 2009 Membrane fusion: role of SNAREs and calcium. *Protein Pept. Lett.* **16**, 712-717.

Jones, R. J., Hoegh-Guldberg, O., Larkum, A. W. D. & Schreiber, U. 1998 Temperature-induced bleaching of corals begins with impairment of the CO_2_ fixation mechanism in zooxanthellae. *Plant Cell Environ.* **21**, 1219-1230. (doi: 10.1046/j.1365-3040.1998.00345.x)

Kloepper, T. H., Kienle, C. N. & Fasshauer, D. 2007 An elaborate classification of SNARE proteins sheds light on the conservation of the eukaryotic endomembrane system. *Mol. Biol. Cell* **18**, 3463-3471. (doi: [0.1091/mbc.E07-03-0193](http://www.molbiolcell.org/cgi/content/abstract/E07-03-0193v1))

Muscatine, L. & Pool, R. R. 1979 Regulation of numbers of intercellular algae. *Proc. R. Soc. B* **204**, 131-139. ([doi:10.1098/rspb.1979.0018](http://dx.doi.org/10.1098/rspb.1979.0018))

Ohya, T., Miaczynska, M., Coskun, U., Lommer, B., Runge, A., Drechsel, D., Kalaidzidis, Y. & Zerial, M. 2009 Reconstruction of Rab- and SNARE-dependent membrane fusion by synthetic endosomes. *Nature* **459**, 1091-1097. (doi:10.1038/nature08107)

Sawyer, S. J. & Muscatine, L. 2001 Cellular mechanisms underlying temperature-induced bleaching in the tropical sea anemone *Aiptasia pulchella*. *J. Exp. Biol.* **204**, 3443-3456. (doi: 10.1241/jeb .009597)

Strychar, K. B. & Sammarco, P. W. 2009 Exaptation in corals to high seawater temperatures: low concentrations of apoptotic and necrotic cells in host coral tissue under bleaching conditions. *J. Exp. Mar. Biol. Ecol.* **369**, 31-42. ([doi:10.1016/j.jembe.2008.10.021](http://dx.doi.org/10.1016/j.jembe.2008.10.021))

Vassilieva E.V. & Nusrat A. 2008 Vesicular trafficking: molecular tools and targets. *Methods Mol. Biol.* 440: 3-14.
